# Poly(lactic Acid): A Versatile Biobased Polymer for the Future with Multifunctional Properties—From Monomer Synthesis, Polymerization Techniques and Molecular Weight Increase to PLA Applications

**DOI:** 10.3390/polym13111822

**Published:** 2021-05-31

**Authors:** Evangelia Balla, Vasileios Daniilidis, Georgia Karlioti, Theocharis Kalamas, Myrika Stefanidou, Nikolaos D. Bikiaris, Antonios Vlachopoulos, Ioanna Koumentakou, Dimitrios N. Bikiaris

**Affiliations:** Laboratory of Polymer Chemistry and Technology, Department of Chemistry, Aristotle University of Thessaloniki, GR-541 24 Thessaloniki, Greece; euagelia226@gmail.com (E.B.); basdanil17@gmail.com (V.D.); geocar1997@gmail.com (G.K.); xkalamas@gmail.com (T.K.); myroforastefanidou@gmail.com (M.S.); nbikiaris@gmail.com (N.D.B.); antwnis97@gmail.com (A.V.); iwanna.koumentakou@gmail.com (I.K.)

**Keywords:** poly(lactic acid), synthesis, melt polycondensation, ring opening polymerization, catalysts, solid state polymerization, chain extension, applications

## Abstract

Environmental problems, such as global warming and plastic pollution have forced researchers to investigate alternatives for conventional plastics. Poly(lactic acid) (PLA), one of the well-known eco-friendly biodegradables and biobased polyesters, has been studied extensively and is considered to be a promising substitute to petroleum-based polymers. This review gives an inclusive overview of the current research of lactic acid and lactide dimer techniques along with the production of PLA from its monomers. Melt polycondensation as well as ring opening polymerization techniques are discussed, and the effect of various catalysts and polymerization conditions is thoroughly presented. Reaction mechanisms are also reviewed. However, due to the competitive decomposition reactions, in the most cases low or medium molecular weight (MW) of PLA, not exceeding 20,000–50,000 g/mol, are prepared. For this reason, additional procedures such as solid state polycondensation (SSP) and chain extension (CE) reaching MW ranging from 80,000 up to 250,000 g/mol are extensively investigated here. Lastly, numerous practical applications of PLA in various fields of industry, technical challenges and limitations of PLA use as well as its future perspectives are also reported in this review.

## 1. Introduction

It has been estimated that until today, global polymers’ total production has reached about 9 billion tons [1]. From these plastics, only 9–10% is recycled and reused, another small amount (12%) is incinerated, while the biggest percentage (78–79%) is accumulated in environment (oceans, lakes, rivers and landfills). Consecutively, they pose serious problems—mostly, for their disposal, environmental contamination, toxicity to the ecosystem and human health. Despite the fact that plastic pollution has become the biggest public enemy today, plastic materials, due to their benefits and advantages over other already used materials, are present almost everywhere in human life. It is reported that their total global production topped 359 million tons in 2018 [2].

Most of them are used as packaging materials, about 35–45 percent of global plastics production, including many single use plastics (SUPs), such as straws, cutlery, cups, bags, packaging films and bottles. Due to their lightness, they cannot be easily recycled, thus contributing to the environmental pollution. The large presence of plastic waste in the natural habitat has a significant effect on the ecosystem due to their huge concentration, widespread distribution, and non-biodegradable properties. Most of them end up in oceans (about 13 Mts per year) where they degrade to microplastics with diameters <5 mm. These microplastics pose a big threat to aquatic and land living organisms [3], while it has been reported that they can pass into the human body via drinking water or the food chain [4,5] with unknown consequences for human health. Plastic islands and microplastics spotted in the aquatic ecosystem are significant evidence of plastic pollution in the environment [6]. Furthermore, environmental carbon dioxide emissions, as well as depletion of fossil resources, are two serious problems concerning the polymers industry.

**Figure 1:**
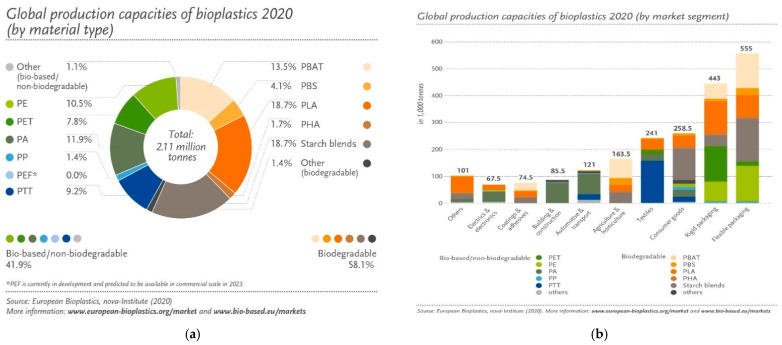
(**a**) Global production of bioplastics 2020 (by material type); (**b**) global production capacities of bioplastics 2020 (by market segment) (https://www.european-bioplastics.org/market, accessed on 27 April 2021) [8].

One solution in combating these problems is to use renewable resources for polymer production (biobased polymers). The worldwide interest in biobased polymers has accelerated in recent years [7]. However, as demand is rising, and with more sophisticated biopolymers such as polyethylene, polypropylene, polyamides, poly(ethylene terephtalate) (PET), furanoate and vanillate polyesters as well as many other aliphatic polyesters [1,2,3,4,5,6], the bioplastics market is continuously growing and diversifying. European Bioplastics in cooperation with the nova-Institute compiled the latest market data of global bioplastics production, which is set to increase from around 2.11 million tons in 2020 to approximately 2.87 million tons in 2025 (Figure 1) [8]. Nevertheless, the research community still focuses on biobased and biodegradable polymers such as PLA, poly(glycolic acid) (PGA) and their PLA-co-PGA copolymers, poly(hydroxyalkanoates) (PHAs) such as poly(hydroxy butyrate) (PHB) and poly(hydroxy valerate) (PHV) and their copolymers, succinate derived polyesters, polycaprolactone (PCL) and others. Biodegradable plastics account for almost 60 percent (over 1.2 million tons) of the global bioplastics especially due to PLA and PHA’s significant growth rates. PHA production increased from 5.3 million tons to 17.0 million tons between years 2013 and 2020, while PLA global production was around 190,000 tons in 2019 (Figure 1), with new investments in the US and in Europe announced every year [6,9].

The development of biodegradable polymers is showcased as one of the most effective innovations in the polymer industry for addressing environmental issues [10]. For this reason, biodegradable polymers are used in various applications, from biomedicine, additive technologies, film, fibers [11], packaging [12,13], automotive to agriculture [14].

PLA is one of the most promising polymers used in these applications [15] and is properly called “polymer of the 21st century “. It is the only one, synthesized on a greater scale that is concurrently: biocompatible, biodegradable and biobased [16]. PLA is an aliphatic biobased polyester derived from lactic acid (2-hydroxypropionic acid) [9,17,18,19], which is mostly derived from animal or plant sources such as cellulose, starch, corn, fish waste and kitchen waste [20]. Carothers in 1932 was the first to synthesize PLA with low molecular weight, while DuPont in 1954 patented a product with a higher molecular weight [17]. PLA exhibits good mechanical strength, biocompatibility, biodegradability and mainly high compostability, [21] constituting it an ideal alternative to the traditional petroleum-based polymers. In addition, it is expected to be widely used in many fields, such as food packaging industry, automotive and medical applications. Today it seems to be the friendliest environmental polymer with ever increasing applications and demands for larger production.

Derived from biomass, PLA is a conventional bioplastic that can be made in a process of three steps including fermentation, separation and polymerization. PLA is a biodegradable polyester polymerized from monomers derived from renewable sugarcane, cornstarch and other abundant natural resources [17]. An intensive large-scale process from recombinant corn which shrinks production costs has been implemented by NatureWorks Co. (Blair, NE, USA) [14]. The typical method for synthesizing PLA is the ring opening polymerization (ROP) of lactide monomer, a process which uses several kind of catalysts such as zinc, tin, lead and aluminum, initiators such as sec-, -n and tert-butyl lithium, and solvents such as diphenyl ether, chloroform and toluene [14]. The molecular weights of the polymer fabricated by the ring opening polymerization can be controlled by the residence time, catalyst type, consternation and temperature. The sequence and ratio of L- and D-lactic acid units in the final polymer could also be controlled [17]. PLA and its degradation products, mostly H_2_O and CO_2_, are neither carcinogenic nor toxic to the human body, hence making it a perfect polymer for biomedical applications including clips, sutures, and drug delivery systems (DDS). In addition, PLA can be formulated by several techniques such as melt extrusion, film casting, blow molding, and fiber spinning due to its greater thermal processability in comparison to other known biomaterials [22].

This review summarizes monomer synthesis of lactic acid and lactide, new synthesizing methods (melt polycondensation and ring opening polymerization) in PLA production introduced over the past few years and attempts to explain their mechanisms. The effects of used catalysts and polymerization conditions on prepared molecular weights of PLA are extensively discussed. In addition, chain extension and solid-state polymerization techniques used for the production of high-molecular weight polymers are also highlighted. Lastly, numerous applications of PLA in medical and biomedical fields, automotive, construction, food packaging, agriculture and single use items production, are also thoroughly presented. Innovations, using advanced PLA-based composites and developed manufacturing methods, have also been mentioned.

## 2. Synthesis of Monomers

### 2.1. Synthesis of Lactic Acid

Lactic Acid (LA) is industrially manufactured through the anaerobic fermentation of agricultural products, such as wheat, potatoes, sugar-beets, corn and sugarcane molasses [23]. Starch and sugars are converted to lactic acid through bacterial fermentation with the use of Lactic Acid Bacteria (LAB) such as *Lactobacillus casei*, *Lactococcus lactis Lactobacillus acidophilus*, *Lactobacillus delbrueckii* subsp. *bulgaricus (Lactobacillus bulgaricus)*, *Lactobacillus helveticus*, *Streptococcus salivarius subsp. Thermophilus. Lactobacillus helveticus* is the most-commonly used *Lactobacillus* stem and gives racemic mixture of LA. It is also possible to produce LA from cellulosic products such as cotton and agricultural waste, by turning lignin, xylan, arabinan and glycan into LA through fermentation [24,25]. Another raw material source for LA production is whey, derived from dairy by-products. Whey is rich in lactose, and lactose can be turned into LA through microbial fermentation [26].

**Scheme 1:**
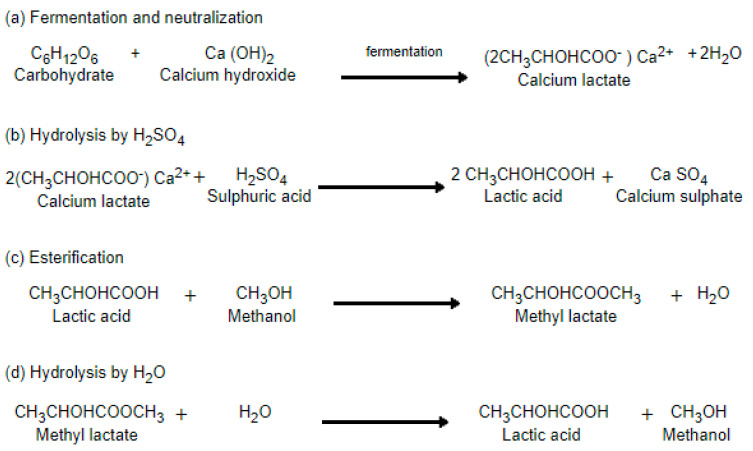
Lactic acid production by fermentation procedure [29].

The industrial production of lactic acid is mostly based on microbial carbohydrate fermentation because it is chemically and economically feasible, when compared with the chemical route [27]. Bacterial fermentation occurs mostly in batch reactors where carbohydrates are converted to LA using water and bacterial cultures in a mixture similar to broth [28]. After the fermentation process, the broth, containing calcium lactate, is filtered to remove cells, carbon treated, evaporated and acidified with sulfuric acid to get lactic acid and calcium sulphate. The insoluble calcium sulphate is removed by filtration; lactic acid is obtained by hydrolysis, esterification, distillation and hydrolysis (Scheme 1) [29]. The fermentation process can also take place in a solid state reactor where there is no water used, but this process is highly unstable since temperature cannot be easily monitored and controlled [30].

Chemical production is also possible by the reaction of acetaldehyde with hydrogen cyanide and hydrolysis of the resultant lactonitrile. Hydrolysis can be performed by hydrochloric acid or sulfuric acid, and thus give ammonium chloride or ammonium sulfate as a by-product. The Japanese company Musashino and Sterling Chemicals Inc in USA are the last big manufacturers that chemically produce lactic acid. Synthesis of both racemic and enantiopure lactic acids is possible from other starting materials such as vinyl acetate or glycerol through catalytic procedures.

### 2.2. Synthesis of Lactide Monomer

Current PLA production is not limited by its polymerization process or the synthesis of lactic acid, but rather than the cyclic lactide synthesis process. This step is the most expensive part of the PLA synthesis scheme and while it is widely studied, there are little to none reports mentioned in the literature. Most lactide synthesis methods are described in outdated patent literature and are based on energy-heavy, non-selective industrial processes and only few are described in literature in recent years [31,32]. According to Pieter Van Wouwe’s review on PLA production from lactide [33], the three most commonly used processes in lactide production are:

(a) The “two-step synthesis of lactide”, where lactic acid undergoes a polycondensation process at 130 °C, resulting in the oligomer lactic acid prepolymer, which is followed by a depolymerization process at temperatures ranging from 150 to 180 °C and low pressure, which gives the lactide dimer ring. Higher temperatures lead to better water removal and lower reaction times, but temperatures higher than 240 °C cause unwanted racemization. The most commonly used catalysts are Sn compounds such as Sn(octanoate)_2_ and SnO, but new and more advanced catalysts such as Pb-Oxides and rare-earth metals are being studied in order to provide better water robustness and minimize racemization. Pb-Oxides are toxic whereas rare-earth metals are not, but the later cause unwanted racemization.

(b) The “gas-phase synthesis of lactide”, a catalytic method in which LA is vaporized and reacted over a (plug-flow) catalyst bed while an inert carrier gas stream is used. Recommended catalysts are SnO and other oxides containing metals from group III, IV, V, or VIII while high-SiO_2_-content SiO_2_–Al_2_O_3_ materials are also suitable. One of the main advantages compared with the traditional two-step process is the faster reaction times which consequently minimize degradation and racemization reactions. Investment costs are estimated to be lower than in the two-step process because this gas-phase process operates at atmospheric pressures instead of very low pressures. Although this process offers very-high yields (70–90%) and low racemization percentages, the reported volumetric productivities are very low to be considered as a viable alternative process.

(c) The “one-step liquid-phase process”, where water removal takes place during the ring-closing reaction and thus lactide is synthesized directly from an aqueous LA solution though condensation rather than through transesterification. This method introduces zeolites as heterogenous catalysts to the bioplastic industry. H-Beta-zeolites are used, resulting in lactide yields of more than 80 % and stereoselectivity is higher than 99%. This means that the unwanted meso-lactide isomer is not produced, which is in contrast to the two-step process.

Synthesis of lactide nowadays is still performed by using an energy-intensive process. The recent growth in patent applications with different methods for alternative lactide production processes proves that a more financially and environmentally attractive method is needed. The main disadvantages in the current industrial two-step process are racemization, low selectivity, and high energy costs. One-step gas-phase methods result in lower racemization percentages, and although high yields can be reached, the volumetric productivity is very low as the feed is highly diluted in an inert gas stream. The most recent one-step liquid phase approach seems to combine the best of both worlds, giving high yields and very-high productivities, while racemization is avoided through the mild conditions and the nature of the catalysts.

## 3. Synthesis of PLA

### 3.1. PLA Synthesis via Polycondensation of Lactic Acid

Since LA is a chiral molecule with D-type and L-type isomers, three forms of polylactic acid are formed: poly(L-lactic acid) (PLLA), poly(D-lactic acid) (PDLA), and poly(D,L-lactic acid) (PDLLA) [22,34].

One of the main origins for the recent extended use of PLA is attributed to the cost-effective production of high-molecular-weight PLA polymers (higher than ∼100,000 Da). These polymers can be produced applying various techniques, including azeotropic dehydrative condensation, direct condensation polymerization, and/or polymerization through lactide formation [35]. By and large, the commercially available high-molecular-weight PLA is produced via the lactide ring-opening polymerization route [34,35,36,37].

Direct polycondensation of lactic acid is mostly carried out in bulk by distillation of condensation water with or without the presence of a catalyst, while vacuum and temperature are gradually increased [22].

**Scheme 2:**
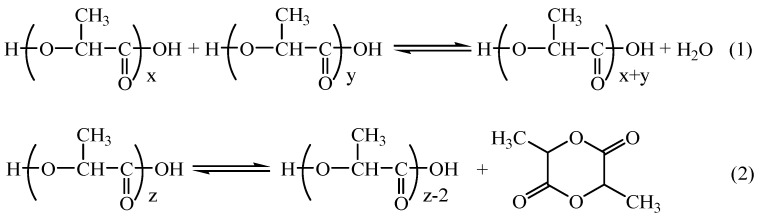
Reactions taking place during poly (lactic acid) synthesis [22].

The polycondensation system of LA involves two reaction equilibria: (1) dehydration equilibrium for esterification and ring-chain equilibrium involving the (2) depolymerization of PLA into lactide (Scheme 2) [22].

The direct polycondensation is a step-growth polymerization reaction where water is removed as by-product [38]. However, the highly viscous reaction mixture, makes it difficult to remove water from it [39] and thus as a result of the unfavorable reaction equilibrium constant and the low reaction rate, direct polycondensation of LA produces only low-molecular-weight PLA (<50,000 g·mol^−1^) [36,39]. To solve this problem there have been many attempts made to produce high-molecular-weight PLA by direct polycondensation of LA or its oligomers [36]. In recent years, azeotropic polycondensation (AP) and solid state polymerization (SSP) are the two main directions [39]. Moreover, the stereoregularity cannot be controlled during polycondensation. The polymer thus possesses inferior mechanical properties. So, this method is employed only if the polymer of low molecular weight is required [22].

In order to obtain a high molecular weight PLA, simultaneous control of dehydration equilibrium and ring-chain equilibrium is highly recommended. Under typical reaction conditions with high vacuum pressures and high temperatures to promote the dehydrative polycondensation, the evaporation of lactide is also favorable for inducing depolymerization. One possible way to promote polycondensation is to activate the dehydrative reaction and deactivate the formation of lactide by the possible selection of a catalyst [22]. In an effort to verify this consideration, Moon et al. [40] executed an intensive screening test to find an sufficient catalyst for the melt polycondensation of LA. In this catalyst screening, it was noticed that the polarity of the reaction system was greatly altered with the progress of the condensation, resulting in an ultimate change in the catalyst activity. The initial LA and its primary condensates contained both hydroxyl and carboxyl groups in high ratio, leading to a high polarity in the reaction system, whereas the resultant PLLA was consisted of less polar ester groups, causing a significant decrease in its polarity. Deterioration of the catalyst activity is mainly attributed to this polarity change. The effects of various catalysts such as metallic and nonmetallic, organic and inorganic ones, and lastly heterogeneous and homogeneous in the polycondensation of PLLA was investigated. Typical results are shown in Table 1 [40]. From the used catalysts (GeO2, Sb_2_O_3,_ ZnO, Fe_2_O_3_, Al_2_O_3_, SnO, SnCl_2_ · 2H_2_O, TiO_2_, SiO_2_) the most effectives were SnO and SnCl_2_ · 2H_2_O given MW 50,000 and 41,000 g/mol, respectively, while the less effectives were TiO_2_ and SiO_2_ producing MW 11,000 g/mol in both cases [41]. However, in all polymerizations extremely long periods of time (ranged from 20 up to 30 h) have been used and temperatures 180–200 °C have been conducted. One additional drawback of melt polycondensation reactions is that with the use of the mentioned catalysts PLA with discoloration is always produced. So, application of catalyst is very important for polymerization and its corresponding product.

Transition metal compounds (e.g., SnCl_2_, ZnO, Fe(Lactate)_3_, Ti(OBu)_4_, etc.) and Brønsted acids (e.g., H_2_SO_4_, p-toluenesulfonic acid, CH_3_SO_3_H, perfluorinated resin, sulfonic acid, etc.) are the mostly used catalysts for polycondensation reaction of PLLA [41]. The disadvantage of using metal catalysts is the contamination of final products, thus constituting them unsafe for biomedical applications and food packaging. Direct polycondensation method catalyzed by Candida Rugosa Lipase catalyst was used so as to synthesize safe PLA for food packaging and biomedical applications [42]. Sulfonic acid-operated task-specific ionic liquids, known as Brønsted acidic ionic liquids (BAILs), stable to vacuum, water, and heat, have displayed excellent catalysis for esterification. BAILs with imidazolium cation and different anions were logically used as catalysts or cocatalysts of stannous chloride dihydrate (SnCl_2_·2H_2_O) for l-lactic acid’s melt polycondensation under nearly high temperature and vacuum. At all times binary catalysts of BAILs and SnCl_2_·2H_2_O exhibited greater catalytic capability in comparison with the single component. In a recent study, the impacts of acidity and ionic structures of BAILs, the amount of catalysts and pressure on polycondensation rate to molecular weight and properties of the produced PLLA, were carefully investigated [41]. High molecular weight PLLA was synthesized at 160 °C, using multiple catalysts and adding them at different times. At first, SnCl_2_·2H_2_O and maleic anhydride were added, and reaction was allowed to run. After 20 h, p-toluene sulfonic acid was added and reaction was allowed to run for a total of 13, 19 and 25 h. The molecular weight of PLA was increased with the lengthening of the reaction time from 38 h to 50 h. The average molecular weight also increased from 49.200 to 55.800 g/mol, whereas the polydispersity index showed an upward course from 5.47 to 14.23 [43]. Furthermore, compared to the traditional binary catalysts, it was found that p-toluenesulfonic acid/SnCl_2_·2H_2_O, BAILs/SnCl_2_·2H_2_O could enhance the thermal behavior of the products [41]. Decompression rates and different polymerization times were reported to be significant factors during polycondensation. Chen et al. [44] synthesized PLA by the direct bulk condensation polymerization using titanium(IV) butoxide as a catalyst at different polymerization times. The duration of the decompression of the reaction pressure to 1 Torr is important when producing high-molecular-weight PLA. When the polymerization reactor was decompressed step by step for 7 h (extremely slowly), the molecular weight of PLA reached as high as ∼130.000 g/mol. The increase of time in the esterification reaction from 3 to 7 h also raised the molecular weight of PLA from 30.000 to 120.000 g/mol [44]. L-lactic acid was polymerized by direct polycondensation (DP) under vacuum without solvents, initiators and catalysts in an effort to decrease the production cost of polylactic acid (PLA). Experiments were conducted at polymerization temperatures (Tp) of 150–250 °C. After 89 h under vacuum at 200 °C the maximum molecular weight of obtained PLA was 90 kDa. Above 200 °C, PLA is subjected to thermal degradation by specific scission [14]. To enhance thermal stability of PLA, Eğri et al., synthesized it by direct condensation polymerization of lactic acid with the presence of microperlite [45]. Furthermore, it was reported that using perlite within the reaction medium resulted to an increase in the molecular weight of the polymer.

In all above described polycondensation procedures, most of the molecules involved in the reaction such as monomers, oligomers, and eliminated molecules such as water, have high polarity and it is reasonable to expect microwave irradiation to accelerate the reaction [36].

The microwave initiated polycondensation is a new approach for PLA production. The time needed for this process is quite less compared to the standard polycondensation process, but the progress and improvement of the molar mass is the most important factor [46]. Nagahata et al. [36] reported the synthesis of PLA with a molecular weight of ≈16.000 g/mol by a single-step direct polycondensation of LA, which can be achieved by effectively using microwave irradiation. They also found that the reaction time can be considerably shortened in comparison to conventional polycondensation at the same temperature. Tin catalysts and the reduced pressure were found to be strikingly effective to obtain polymers with a molecular weight higher than 10.000 g/mol. Among the catalysts tested by Nagahata’s group, the SnCl_2_/p-TsOH binary catalyst exhibited the highest activity. The obtained polymer is colorless and has enough strength to make pellets and films, plus it can be applied as raw material of biodegradable plastics [36]. Shahrul et al. [38] studied also the case of performing polycondensation of LA to PLA assisted by microwave in the absence of a catalyst. The increase of microwave power was found to result in a higher mass loss mostly likely due to the extensive evaporation of water molecules and the intensified polycondensation reaction [38].

Azeotropic distillation is one of the most promising alternatives to the direct polycondensation of LA. By this method, the water produced as by-product during the polycondensation is removed efficiently by using an appropriate azeotropic solvent. In 1994, Mitsui Toatsu Chemicals patented an azeotropic distillation method using a high-boiling point solvent to force the removal of water to the direction of esterification process for the fabrication of high molecular weight PLA. The control of the equilibrium between monomer and polymer can be tuned in the chosen organic solvent to produce PLA with comparatively high molecular weight in a single-step procedure. The temperature of the polymerization is lower during azeotropic distillation, than the polymer melting point, which prohibits the presence of impurities derived by depolymerization and racemization. In this process, the selected solvent is vital for the polymerization conditions and the properties of the final PLA [37]. Azeotropic distillation has been used by Maryanty et al., to produce PLA using lactic acid product from rice straw fermentation. Xylene solvent was used to absorb water, and SnCl_2_ catalyst was used to accelerate the reaction. The polymerization process was carried out for 30 h [47].

### 3.2. Ring—Opening Polymerization of Lactide

#### 3.2.1. Mechanisms of Ring-Opening Polymerization

##### 3.2.1.1. Coordination–Insertion Mechanism

Polycondensation reactions as reported previously lead, in almost all cases, to low molecular weights PLA. A common way for synthesizing high molecular weight PLA is coordination-insertion ring-opening polymerization (ROP) of cyclic lactide diesters [48]. ROP is executed typically in the presence of metal carboxylate with a hydroxyl-containing compound, normally alcohols, H_2_O, or macromolecules with hydroxy end group (denoted as ROH). Poly(ethylene glycol) (PEG) is one of the most common hydroxyl-containing chemicals in assisting synthesis of polyesters, and PEG itself is a hydrophilic polymer which has been widely applied as medical excipient and bio-matrix [49,50,51].

**Scheme 3:**
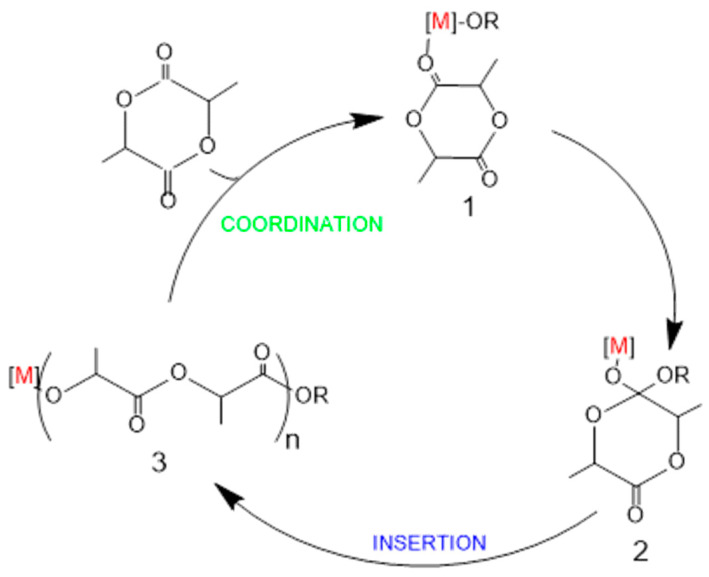
Coordination– insertion mechanism for ROP of lactide Reprinted with permission from ref. [55]. Copyright 2017 Springer International Publishing AG.

Many well-defined transition metals and main cluster metal catalysts proceed through a coordination–insertion mechanism for the ring-opening polymerization of lactide [52]. The golden customary reaction to obtain aliphatic polyesters is ROP of cyclic esters power-assisted by both ROH and Tin(II) bis(2-ethylhexanoate) (stannous octoate, Sn(Oct)_2_). This mechanism is also regarded to work in the cases of many other transition metal, main group metals, and lanthanide complexes such as Al, Mg, Zn, Fe, Ti, Sm and Ln [53,54]. In this mechanism, the electrophilic activation of the lactide for attack by the nucleophilic alkoxide group on the metal is caused by coordination of lactide by a Lewis acidic metal, frequently a metal alkoxide (e.g., 1, Scheme 3). This results in formation of an intermediate that is similar to the tetrahedral intermediate commonly observed in organic chemistry during the interconversion of carboxylic acid derivatives (e.g., 2, Scheme 3). Then this intermediate collapse and gives a reformed alkoxide that incorporates a unit of lactide, thus aiding the continuation of ROP. Propagation occurs by subsequent lactide coordination and alkoxide insertion until the metal–alkoxide bond is cleaved by termination reactions. Consequently, PLA with an ester end group derived from the initiator is obtained.

The first experimental sign of a coordination–insertion mechanism for the ROP of lactide was reported by Kricheldorf et al. [56] and shortly after by Jerome and coworkers [57]. These research groups characterized the obtained polymer by ^1^H-NMR and ^13^C-NMR spectroscopies and IR spectroscopy, confirming that the lactide ring opens via ester cleavage and the insertion of the lactide monomer into the metal–alkoxide bond.

##### 3.2.1.2. Activated Monomer and Activated Chain Mechanisms

**Scheme 4:**
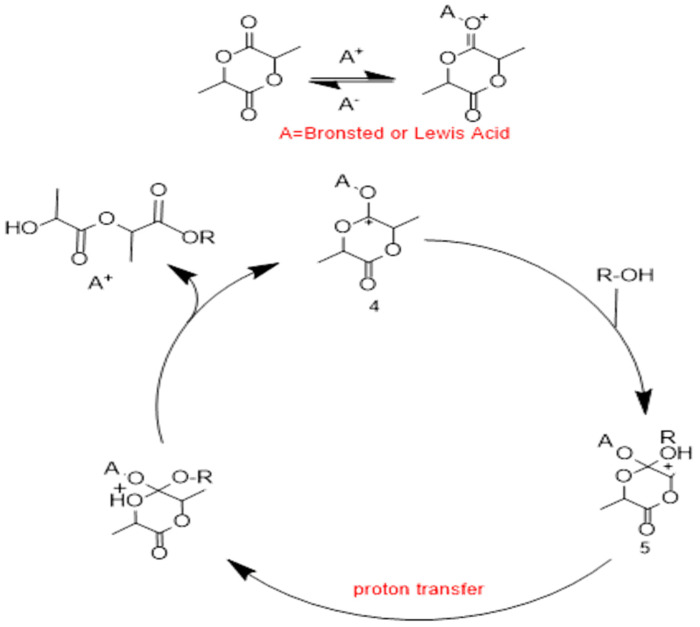
Activated monomer mechanism for ROP of lactide Reprinted with permission from ref. [55]. Copyright 2017 Springer International Publishing AG.

Aside from the coordination–insertion mechanism, another frequent route proposed for lactide polymerization reactions is the activated monomer mechanism (AM) (Scheme 4) [58,59,60], which is a convenient way using metal catalysts [61,62]. The activated chain end mechanism (ACE) can be also applied to many ROP processes including cationic, anionic, organo-catalytic, psedo-ionic and applies to miscellaneous heterocycles, such as oxiranes, lactones, cyclic phosphoesters, carbonates, etc. Both industrial and academical research groups in order to boost the properties of PLA develop efficient catalytic systems for the preparation of PLA. In a recent research, Mandal and Chakraborty [63] investigated the potential use of low-cost Co_2_O_3_ and MnO_2_ as catalysts for the ROP of rac-lactide (rac-LA). The polymerization continued in a controlled way with the formation of high molecular weight (Mn) and narrow dispersity (Ð). MALDI-TOF and ^1^H NMR analyses of low Mn oligomer from rac-LA indicated that the polymerization was followed by an activated monomer mechanism.

This process initiates with electrophilic activation of lactide using either a Bronsted acid initiator or a Lewis acidic catalyst. Either way, an oxo-carbenium ion intermediate 4 is formed that acts as the activated monomer poised for nucleophilic attack. At that point, either the initiator or the growing polymer chain attacks this oxo-carbenium ion, forming the tetrahedral intermediate 5. After proton transfer, collapse of the intermediate results in ring opening and incorporation of a lactide unit into the growing polymer chain. In addition to traditional Lewis acids and Bronsted acid catalysts, the activated monomer mechanism has also been proposed for guanidine-type organo-catalysts where hydrogen bonding to the monomer is thought to be the primary source of monomer activation [64]. The AM mechanism competes with the ACE mechanism. Usually the choice of mechanism depends on both the kinetic data and the value [monomer]/[OH] [60].

##### 3.2.1.3. Nucleophilic Activation of the Polymer Chain End

**Scheme 5:**
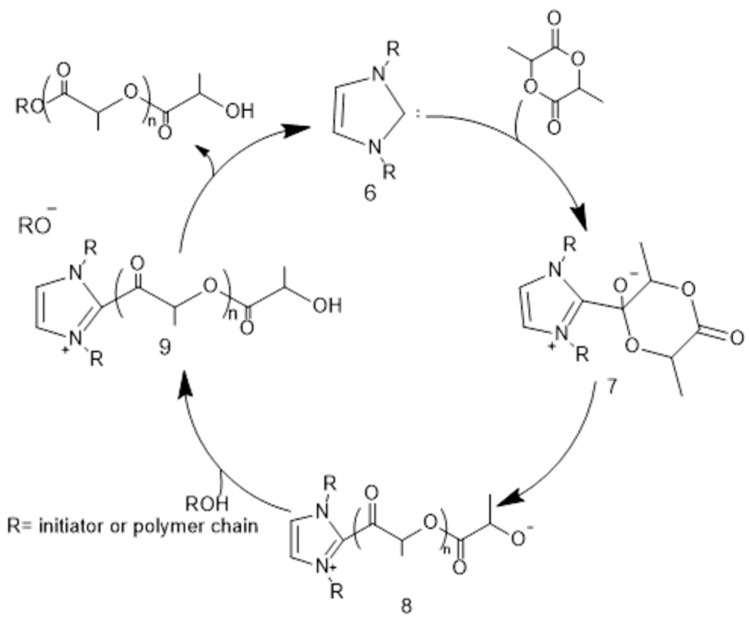
Ring-opening polymerization of lactide by a mechanism involving nucleophilic activation of the polymer chain end in the presence of an N-heterocyclic carbene catalyst Reprinted with permission from ref. [55]. Copyright 2017 Springer International Publishing AG.

A third mechanism for acquiring high molecular weight PLA is through nucleophilic activation of the polymer chain end. Nucleophilic activation is one the most efficient way to homopolymerize DL-LA and was consequently used for synthesizing the first PDLLA block with terminal OH groups. Nucleophilic catalysts, such as N-heterocyclic carbenes (NHCs, e.g.,6, Scheme 5) and 1,5,7-Triazabicyclodec-5-ene (TBD) [65] as a guanidine type molecule are frequently applied to catalyze the ROP of lactide by this mechanism [63,66,67]. The free carbene functions as a nucleophile, which attacks the carbonyl group of the lactide to form the zwitterionic intermediate 7. Collapse of this intermediate results in ring opening and the formation of a new zwitterionic intermediate 8. After, this intermediate is deprotonated by the alcohol additive, which forms an alkoxide that attacks the acylium ion intermediate 9 to create a second zwitterionic tetrahedral intermediate. NHC catalyst is emerged by the collapse of this intermediate. Chain propagation occurs by subsequent nucleophilic activation of a second equivalent of monomer and capture by the alcoholic end group obtained from the polymer initiation. The polymerization propagates in this way till all of the lactide monomer quantity is consumed.

### 3.3. Catalytic Systems of ROP

The ring-opening reaction mechanism of lactides involves five different kinds of catalysts such as: metal catalysts, organic catalysts, cationic catalysts, anionic catalysts and stereo-controlled polymerization.

#### 3.3.1. Cationic Catalysts

Cationic ring-opening polymerization is a process where positive factors are used to open the rings. The polymerization of L,L-lactide catalyzed by strong protic acids in the presence of diols leads to polymers terminated at both ends with hydroxyl groups. In such process the strong acid acts as catalyst and the –OH group containing compound as initiator. This method provides many important industrial polymers such as 1,3,5-trioxane and oxirane or 1,3,5-trioxane and 1,3-dioxolane, polytetrahydrofurans, copolymers of tetrahydrofuran and oxirane, poly(3,3-bis(chloro-methyl)oxetanes), polysiloxanes, polymers of ethyleneimine and polyphosphazenes [68]. Τwo main mechanisms are reported in the literature: the cationic polymerization of oxygen-containing compounds is carried out either by an ACE or by an AM mechanism.

In all recent research it has been accurately noted that cationic polymerization of L,L-lactide follows the AM mechanism. However, this occurs even at very low concentrations of the monomer in the earlier stages. For this purpose, very strong organic acids are used as catalysts. However, more attention is being paid in selecting the most suitable initiator. Since the late 1980s, Kricheldorf and his team have reported the characteristic ability of an acid to act as an initiator with great success [69].

**Scheme 6:**
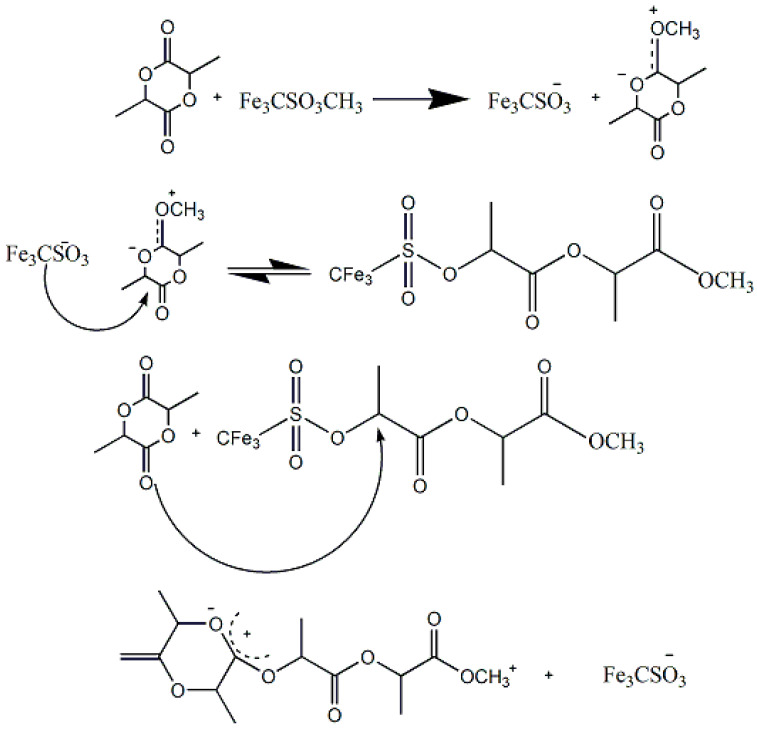
Cationic ROP of LA for PLA synthesis Reprinted with permission from ref. [71]. Copyright 2000 Springer International Publishing AG.

They found out that trifluoromethanesulfonic acid –HOTf (triflic acid) and methyl trifluoromethane sulfonic acid -MeOTf (methyl triflate) could effectively induce the polymerization of lactide, among many others [35] (Scheme 6). The initial process is that the ring of the lactide, activated by receiving acid proton, is opened by the attack of alcohol carbonyl, and lactyl alcohol is obtained. HOTf activates oxygen of lactide so that the alcohol can nucleophilically attack, cut acyl group and catalyze ring-opening reaction [70]. The polymerization proceeds via triflate ester end-groups instead of free carbenium ions, which yields, at low temperatures(<100 °C), an optically active polymer without racemization [71]. Additionally, according to ^1^H-NMR, polymers with methyl ester end groups were obtained with MeOTf as the initiator [69].

In addition, the researchers tested the effects of solvents on polymerization. The polymerization rates were significantly higher in nitrobenzene than in chlorinated solvents [69]. Malgorzata Basko [72] studied the behavior of PLA in CH_2_Cl_2_ at room temperature. Different initiation systems have been used while controlling LA conversion based on optical rotation. It was found that at given conditions only CF_3_SO_3_H (TfA) initiated relatively fast polymerization (~99% in 17 h). Polymerization proceeded also (although much more slowly) with CF_3_SO_3_CH_3_(~99% in 30 days) [72].

Bourissou et al. [73] also exhibited the mixture of trifluoromethanesulfonic acid (as a catalyst) and a protic reagent (such as water, 2-propanol, or 1-pentanol as an initiator) efficiently initiates the cationic polymerization of lactide CH_2_Cl_2_ solution at room temperature. Polylactides (PLAs) with molar masses up to 20.000 g/mol were obtained via an activated-monomer mechanism. HOTf is the only catalyst active in CH_2_Cl_2_ at room temperature, the complete polymerization of 10 equiv of lactide requiring 150–180 min with 1 equiv of water, 2-propanol, or 1-pentanol [73].

#### 3.3.2. Metal Catalysts

**Figure 2:**
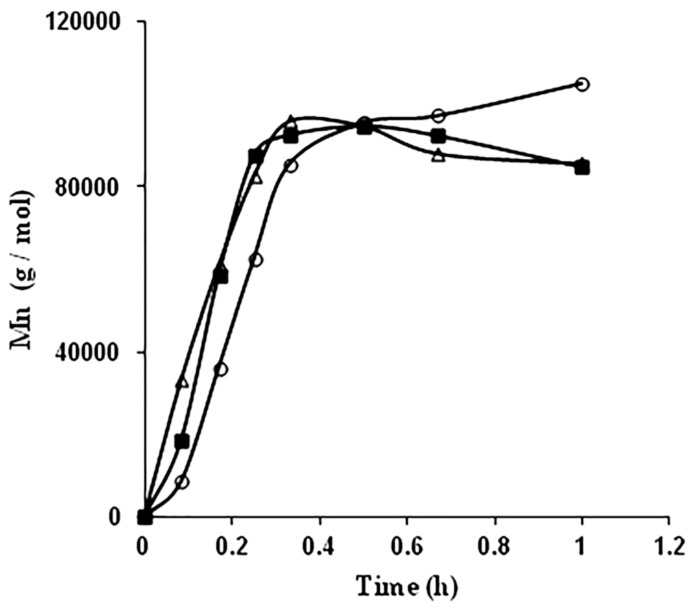
Plot of Mn vs. time at for L-lactide polymerization at different temperatures: o—150 °C; ■—160 °C; ▲—180 °C [74].

Metal complexes of Al, Mg, Zn, Ca, Sn and Zr are extensively used as catalysts for ROP of lactides. Strong bases such as metal alkoxides have also been applied with inadequate success. Stannous(II) 2-ethylhexanoate (Sn(Oct)_2_)is one of the catalysts most widely used as a compound that starts the ring opening polymerization (ROP) of various lactones and lactides [70]. Tin catalyst is easily soluble in lactide monomer and subsequently allows almost complete conversion. It has also low risk of racemization and can be easily removed from the obtained polymer. Even with different mechanisms, this catalyst has reduced nucleophilicity. An alcohol is also necessary to be added during the ROP, which act as initiator and co-catalyst, thus enhancing the rate of reaction, and acts also as an end capping reagent [74]. Karidi et al. [75] studied the polymerization of L,L-lactide with Sn(Oct)_2_ as initiators and co-initiators with different number of hydroxyl groups (i.e., 1,4-butanediol, glycerol, di-trimethylol-propane and polyglycidol). The final products were branched or star shaped polymers. Metkar et al. [74] investigated the polymerization of lactide with 1-pyrene butanol as co-catalyst at 150 ℃ and with different co-catalyst to catalyst ratios of 10, 17, 30. Additionally, they used fixed co-catalyst to catalyst ratio of 17 at the temperatures of 150, 160 and 180 °C. The graphical representation of the molecular weight in terms of polymerization time at different temperatures is interesting (Figure 2).

**Scheme 7:**
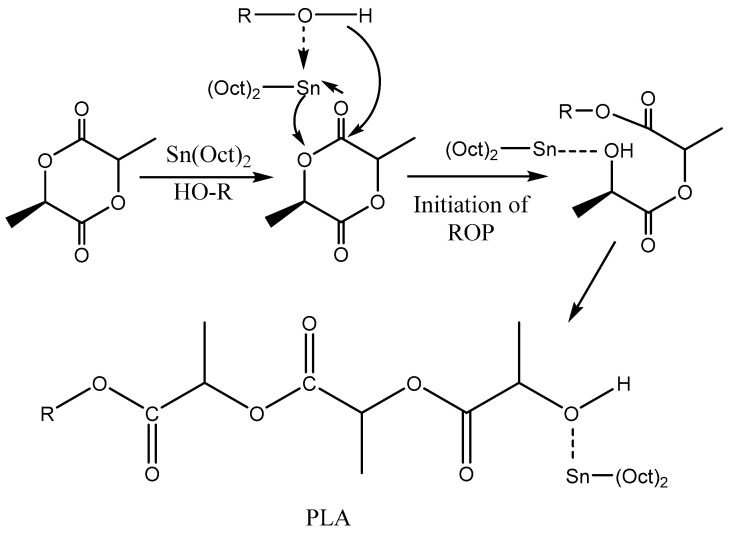
Reaction mechanism for PLA synthesis.

The mechanism of PLA polymerization is very simple and plain. Alcoholic groups of hydroxyl compound act as initiators in ROP of lactides since they react first with Sn(Oct)_2_, to generate a tin alkoxide bond by ligand exchange. Simultaneously, one of the carbonyl groups of lactide interacts temporary with Sn atom. This coordination increases one the hand the nucleophilicity of the formed alkoxide and on the other the electrophilicity of the lactide’s carbonyl group. So, during this coordination there is a rearrangement of the electrons and is much easier the acyl-oxygen bond of lactide dimer to be broken and lactide monomer to be opened (Scheme 7). The alcoholic initiator is covalently bonded with the opened lactide and deactivated. On the other side of initiated monomer there is a new hydroxyl formed, which with the help of Sn(Oct)_2_ can continue the initiation reactions of new lactide monomers and lead to increased MW of PLA chains [58]. Apart from their chain initiating role, the hydroxyl groups can also act as chain transfer agents. As a result, the ROP of L,L-lactide is very sensitive to the hydroxyl groups concentration. Thus, it is of paramount importance to effectively control their concentration, since they largely affect the polymerization rate and molecular weight of PLLA. The MW was calculated about 1.5 × 10^5^ and Mn was about 8 × 10^4^.

In a recent research, PLA was synthesized by ROP using samarium (III) acetate (SmAc_3_) and amino acid complexes of samarium (III) such as l-aspartic acid, l-glutamic acid and glycine. Polymerization reaction temperature was 125 °C and PLA with average-number molar mass of 1.50 × 10^3^–10^4^ was obtained based on a coordination–insertion mechanism [76]. A feature common of many polymerizations that proceed through a coordination–insertion mechanism is the excellent control over polymer molecular weight.

#### 3.3.3. Anionic ROP

**Scheme 8:**
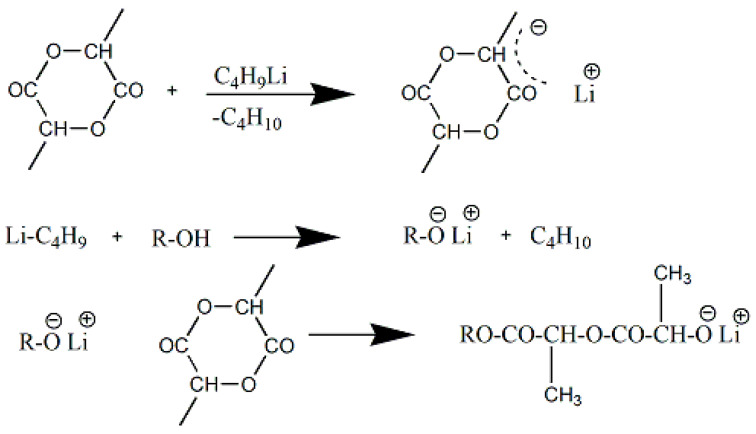
Anionic ROP of LA for PLA synthesis Reprinted with permission from ref. [77]. Copyright 1993 Hüthig & Wepf Verlag, Basel.

The anionic polymerization of lactide has gained little interest until today. Kleine [77], was the first who reported that various strong bases (including LiA_1_H_4_) initiate the anionic polymerization of L-lactide in toluene solution (Scheme 8). Kricheldorf et al. [56] found that initiators of higher nucleophilicity are required for initiating the lactide. Weaker bases, such as potassium benzoate, potassium phenoxide, or zinc stearate, do not act as initiators at low temperatures, rather than high temperatures (120 °C) [71]. They found out that only tert-BuOK and BuLi are effective initiators, but molecular weight was low and fast racemization occurred.

**Scheme 9:**
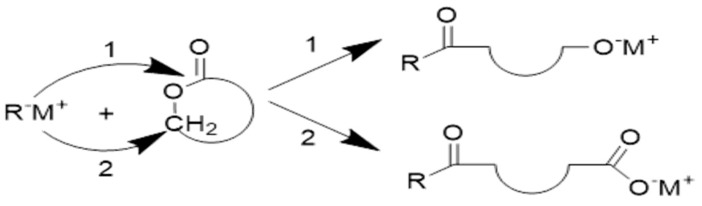
Ring-opening of monomer by (1) acyl- oxygen bond cleavage and (2) alkyl-oxygen bond cleavage Reprinted with permission from ref. [78]. Copyright 2002 Hüthig & Wepf Verlag, Basel. Springer-Verlag Berlin Heidelberg.

Anionic ring-opening polymerization of cyclic ester monomers takes place by the nucleophilic attack of a negatively charged initiator on the carbonyl carbon or on the carbon atom adjacent to the acyl-oxygen, resolving in a linear polyester (Scheme 9). The propagating species are negatively charged and counter-balanced with a positive ion [78].

Scientists argue that the need to find metal-free catalysts stemmed from the great inability of polymers to be used later in biomedical applications. This problem may be overcome by the application of anionic initiatiors based on sodium or pottasium ions, which participate in metabolism of living orgasms and their removal from polymers prior to their use isn’t neccesary. Zhu et al. [79] synthesized PDLA by anionic polymerization in THF at room temperature. The initiator was choosen to be potassium PEG-200. They noticed that the molecular weight of polymer decreased with increase of the amount of initiatior. This may be explained by the degree of transesterification increased at large concetration of initiatior due to its strong basicity. Nontheless, the initiator system was proved to be effective [79].

### 3.4. Enzymatic ROP

**Scheme 10:**
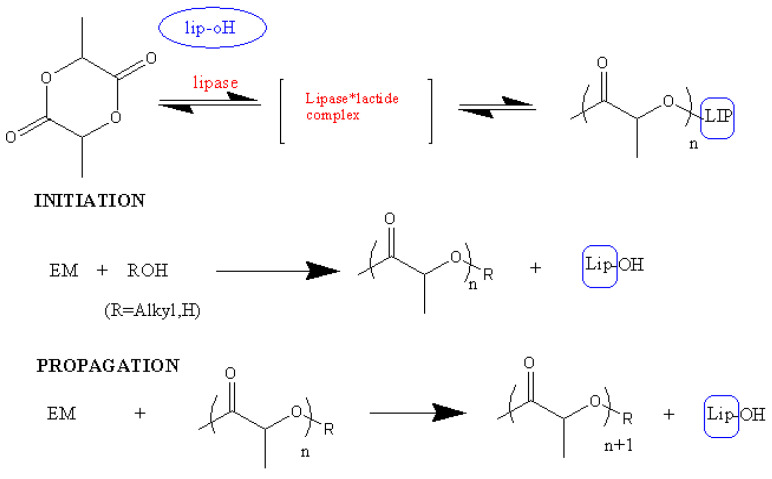
Mechanism of lipase ROP.

Since the first report of enzymatic ring-opening polymerization (eROP) back in 1993 (eROP of CL and VL by lipase), various lactones have been polymerized by enzymatic catalysis to give the corresponding polyesters (Scheme 10). There are several common routes for ring opening polymerization of lactides but each of them has some drawbacks. Enzymatic ROP of lactides using lipases and esterases represents a ‘greener’ alternative to metal-based catalysts [80]. This route can produce PLA that has better physical and chemical properties, and is an eco-friendly method that can be performed under mild conditions [70]. In addition, different strains of enzymes that can execute this process have been studied. The first interesting class of enzymes is lipases. Lipases are esterases which can hydrolyze triglycerides (or esters) to glycerols and fatty acids in a water—oil interface. In nature these enzymes participate in the degradation of food and fats using their hydrolytic ability.

The action mechanism of enzymes is rapid: At first, lactide binds to the enzyme and the ring opening is initiated; an active acyl-enzyme intermediate (EM–enzyme-activated monomer) is formed. The initiation of the polymerization is facilitated by the nucleophilic attack of water molecules into the acyl carbon of the EM. In the following propagation of the polymer chain, the terminal hydroxyl group of the growing polymer chain nucleophilically attacks the EM (Scheme 10).

When it comes to lactides, where the reaction is solvent –free, the system requires a temperature above their melting point e.g., 100–130 °C [81]. Omay et al. developed a new methodology for synthesizing high molecular weight polylactic acid via enzymatic ROP method using free enzyme and Candida antarctica lipase B (CALB) immobilized into chitin and chitosan. Chitin and chitosan were used as organic supports. The average molecular weight of polymer obtained was more than 4 × 10^4^ g/mol. After immobilization, as expected there was a loss of about 15% in activity in the case of enzyme chitosan and 25% in the case of enzymechitin [82]. Rahmayetty et al., used a different lipase, Candida rugosa (CRL), which is a table enzyme, and its use is relatively widespread in the field of biotechnology, especially in hydrolysis reactions, esterification, transesterification and enantioselective biotransformation. The ring-opening polymerization reaction was conducted at various temperatures (70, 90, 110 and 130 °C) and CRL concentrations (1, 2, 3% *w*/*w*)). They noticed that the system has the highest CRL activity at 90 °C, concentration 2% and the polymerization occurred only in the presence of the catalyst. The crystallinity and melting point of PLA were 31% and 120 °C, respectively [83]. However, there are problems that need to be addressed so that the enzyme can completely replace a metal catalyst. Specifically, the recovery of PLA after the reaction, the interaction of lipase with the solvent, and any potential mistakes in measurement of the conversion by titration [84].

## 4. Molecular Weight Increase of PLA

### 4.1. Solid State Polycondensation

Melt polycondensation or ring opening polymerization reactions do not produce PLA of high molecular weight required for several applications. To overcome this limitation, an additional procedure called solid state polycondensation (SSP) is used [85]. SSP is a process in which the polymer chain lengths are increased, and higher molecular weight is obtained through heat in the absence of oxygen and water, by means of either vacuum or purging with an inert gas. SSP process is caried out above glass transition temperature (T_g_) and under melting temperature (T_m_) of PLA and the reaction is driven by temperature, pressure, and the diffusion of by-products from the interior of the pellet to the surface. The molecular weight is raised up in the amorphous religions of a semi-crystalline polymer. Meanwhile, crystalline regions retain their geometrical shape. By searching the literature, it is clear that no significant progress of SSP of PLA during the last 10 years has been achieved and only few papers have been published [86,87,88].

**Figure 3:**
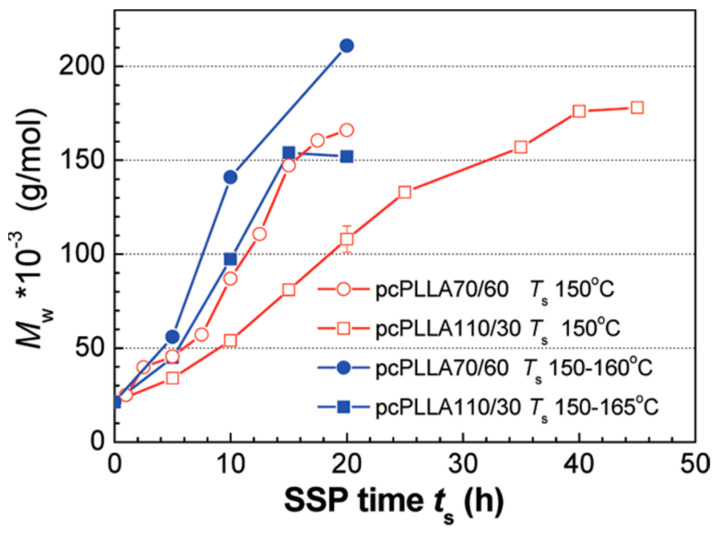
MW growth during SSP at constant and step-raised temperatures for pcPLLA70/60 and pcPLLA110/30, respectively: (○) 70 °C/60 min + 150 °C/20 h; (□) 110 °C/30 min + 150 °C/40 h; (●) 70 °C/60 min + 150 °C/5 h + 155 °C/5 h + 160 °C/10 h; (■) 110 °C/30 min + 150 °C/5 h + 155 °C/5 h + 160 °C/5 h + 165 °C/5 h Reprinted with permission from ref. [89]. Copyright 2012 American Chemical Society.

The effects of pre-crystallization temperature and time as well as SSP temperature on the solid state polycondensation have a significant impact on obtaining high molecular weight PLA. According to Peng et al. [89], the ideal pre-crystallization process is isothermal heat at 70 °C for 1 h, where PLA reaches 45% crystallinity. In this case, slower crystallization rate and smaller crystal sizes have been observed. These crystals continue to expand during SSP, and develop into more perfect ones, due to the continuous increase in crystallinity and melting temperature. The esterification reactions, during SSP specifically, take place in amorphous phase, as the mobility of the chain end groups is higher. In SSP, temperature plays an important role in enhancing the process of molecular weight increase. Progressing SSP at 150−160 °C for 20 h using Sn(Oct)_2_ as catalyst, molecular weight of PLA was increased from 21.300 to 200.000 g mol^−1^ (Figure 3). When the precrystallization time is 110 °C instead of 70 °C, the molecular weight increase is lower. So, it seems that the initial degree of crystallinity reached by preheating is very crucial since it affects the produced molecular weight during the SSP process.

**Figure 4:**
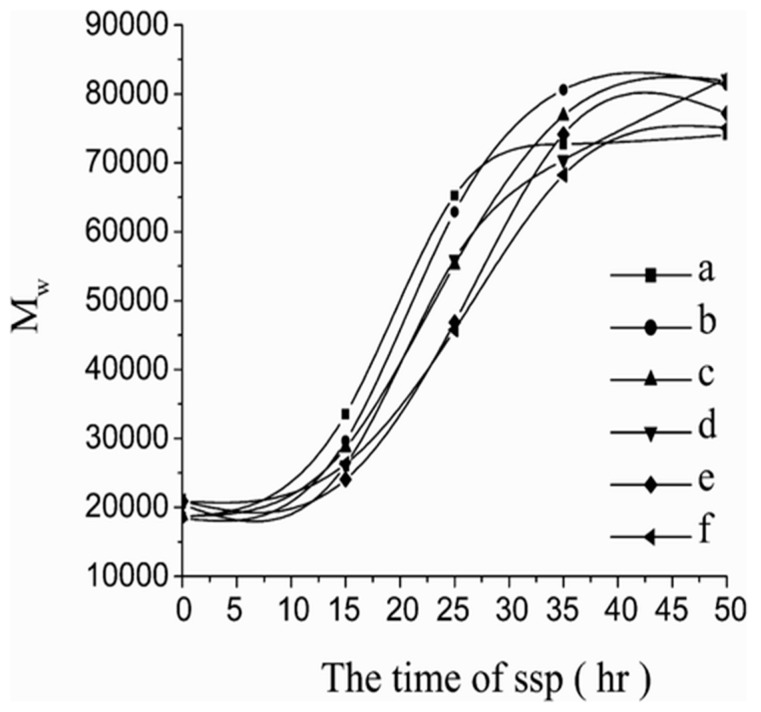
The molecular weight of PLLA prepolymers crystallized for various times as a function of SSP time (**a**) crystallized for 15 min, (**b**) crystallized for 30 min, (**c**) crystallized for 45 min, (**d**) crystallized for 60 min, (**e**) crystallized for 75 min, and (**f**) crystallized for 90 min Reprinted with permission from ref. [90]. Copyright 2006 Taylor&Francis.

Xu et al. [90] used also such higher pre-crystallization temperature and PLA samples were preheated at 105 °C at different times each. SSP process took place at 135 °C under vacuum for various lengths of time. In all samples MW was significantly increased at all crystallization times. Specially, pre-crystallization time of 30 min at 105 °C and later SSP time of 35 h using Tin II chloride dihydrate (SnCl_2_ · 2H_2_O) as catalyst leads to the maximum value of the molecular weight, which was increased by four times (Figure 4). Continuing the process for five more hours, the molecular weight of PLA increased very slowly and even decreased. This is occurring due to the degradation of the macromolecular chains at high temperatures for longer time.

Except of pre-crystallization and SSP temperatures, the used catalyst systems, can also affect the final molecular weight. Some of these catalyst systems are: Tin (II)chloride SnCl_2_, Tin(IV) oxide (SnO_2_), Tin(II) 2-ethylhexanoate (Sn(Oct)_2_) C_16_H_30_O_4_Sn, citric acid anhydrous (CA) C_6_H_8_O_7_, sulfuric acid H_2_SO_4_, p-toluenesulfonic acid monohydrate (pTSA), etc. According to Pavel Kucharczyk et al. [91], using 0.5% of H_2_SO_4_ high MW have been obtained, from 44.000 to 82.600 g/mol progressing within just 6 h. This type of catalyst is a very strong proton acid and exhibits superb performance when used for this purpose. By increasing the amount of catalyst to 1%, a noticeable increase in MW was mentioned reaching 39.200 and 64.500 g/mol after 6 h and 24 h, respectively. Using SnCl_2_·2H_2_O+pTSA (1:1) and Sn(Oct)_2_ catalysts, lower MW have been prepared, while SnO_2_ and CA didn’t show any favourable effect on MW. Katiyar et al. [92] studied also the effects of Sn(Oct)_2_ and Zn(OLLA)_2_ catalysts on MW increase during SSP of PLA. Progressing in different conditions, time, surface morphology of the sample and the same catalyst, received various molecular weights. SSP have been carried out both under vacuum as well as under preheated nitrogen gas sweep at 150 °C with flow rate for 10 h. Progressing at 150 °C under vacuum for 5 h using Sn(Oct)_2_ as catalyst and using PLA as powder, the molecular weight was raised from 26.000 to 100.000 g/mol. Progressing for 5 more hours, it can be reached to 228.000 g/mol. Processing in the same matter, but under nitrogen gas, the reached molecular weight was lower (112.000 g/mol) in 12 h. On the other side, PLA synthesized by Zn(OLLA)_2_, being in powder and under vacuum, has introduced a much lower enhancement of MW indicating that is not such a reactive catalyst as Sn(Oct)_2_.

SSP can take place also between mixtures consisted of different stereoregular kinds such as PLLA and PDLA [93]. The most significant part of this process is the enhancement of mechanical and thermal properties compared with those of PLLA. In this case, PLLA and PDLA polymers have been polymerized separately with specific tasks and later have been triturated. According to Fukushima et al. [94], a 1:1 powder blend of PLLA and PDLA prepolymers having medium molecular weights was used. For pre-crystallization, the sample is annealed at 115 °C for 2 h while due to the reduction of T_m_, the SSP process was performed at a lower temperature than in PLA. A stereocomplex with significantly deteriorated crystallinity was created.

### 4.2. Chain Extenders

**Scheme 11:**
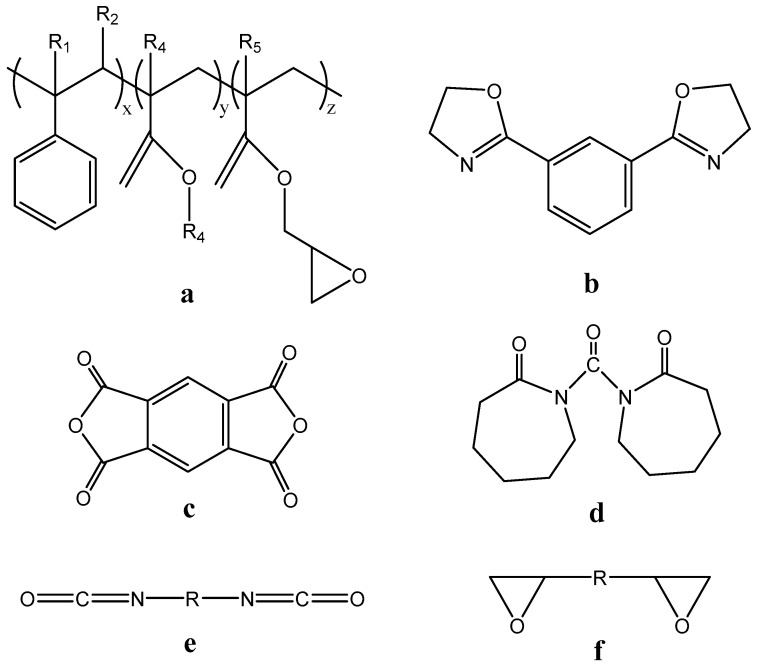
Chemical structure of chain extenders: (**a**) Joncryl^®^ ADR 4368 copolymer produced by BASF Co, (**b**) 1,3-phenylene-bis-2-oxazoline, (**c**) pyromellitic dianhydride, (**d**) 1,10-carbonyl bis caprolactam, (**e**) diisocyanate and (**f**) diepoxide.

Poly(lactic acid) (PLA) is susceptible to severe thermal degradation when processed at high temperature, especially above 200 °C. An unfavorable molecular weight reduction and weight loss induced by both hydrolysis and depolymerization reactions results in poor mechanical performance of obtained products. SSP is the most used technique for increasing its molecular weight. One additional way is the chain extension technique, which was used from many years ago in polyesters [95,96,97,98]. According to this, low molecular weight bifunctional compounds or oligomers with various kinds of functional groups, such as epoxy, anhydride, isocyanates, bis cyclic-imino-esters, bislactams, oxazolines, etc., (Scheme 11) can react with -OH end groups of PLA macromolecules during melt mixing procedure [99,100]. The molecular weight is increased by connecting two polymer chains together, while the degradation and hydrolysis reactions are minimized.

Among the classic chain extenders (CE) the most used one is Joncryl^®^ ADR 4368 produced by BASF Co, which is an oligomer (Mn = 3000 g/mol) of styrene-acrylic copolymer with epoxy groups (epoxy equivalent weight 285 g/mol) (Scheme 11) and T_g_ = 54 °C.

**Figure 5:**
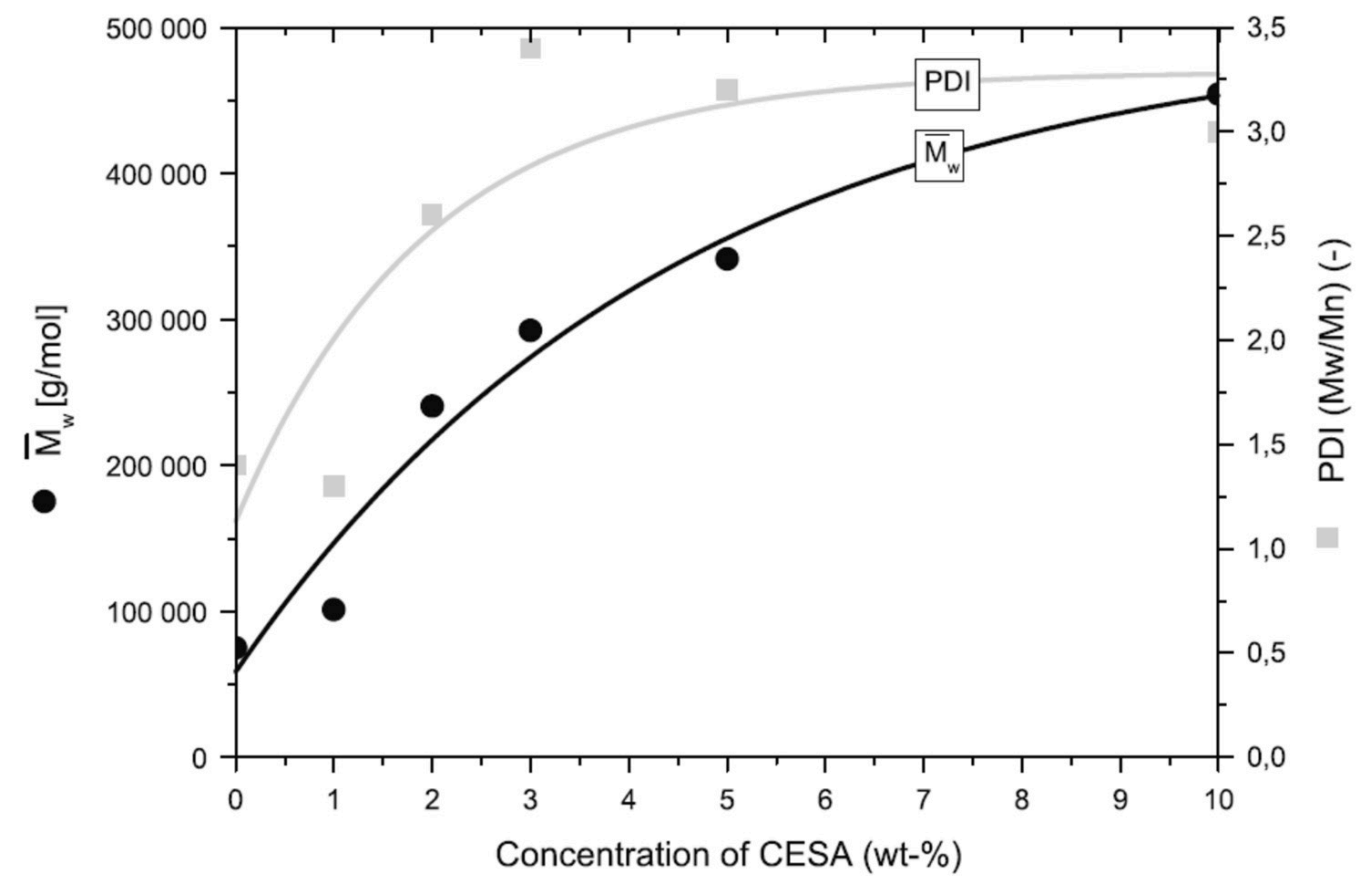
Weight average molecular mass and the polydispersity of PLA with CESA [101].

It was reported that the addition of Joncryl (CESA) causes a significant increase to the molecular weight of PLA and polydispersity (PDI), which both are directly depended from the used CE amount (Figure 5) [101]. According to Elhassan et al., the addition of Joncryl at a ratio of 0.5 wt% and 0.9 wt% results in the enhancement of PLA’s mechanical properties [102]. Before blending the polymer with the CE, PLA pellets must be dried at 70 °C for 48 h so that any moisture is removed. The addition of CE is taking place in a twin-screw micro-compounder at 180 °C and 300 rpm. DSC data revealed a slight increase in T_g_ and T_m_ and a simultaneous decrease in crystallinity with the increase of CE amount.

Due to the molecular weight increase and branching reactions that take place, a significant decrease to the MFI of PLA was reported from the addition of Joncryl. The melt strength increase promotes the formation of PLA foams with fine cellular structure, since it was found that a large number of small cells can be produced by the addition of 1.0 and 1.5 wt% of Joncryl [103,104]. Joncryl and Pyromellitic dianhydride (PMDA) chain extenders can be used together as a solution to the poor melt properties of PLA [105]. The results showed that the chain extension reactions lead to an increase in the storage modulus, complex viscosity, and molecular weight due to the formation of long chain branched structures. The chain extension reaction can take place at 200 °C for 300 s leading to significant increase in the mixing torque as well as PLA’s melt viscosity and molecular weight, but also to reduced crystallinity [106].

The use of other CEs with methacrylate or epoxy groups have been also reported. The effects of acrylated epoxidized soybean oil (AESO) addition on the mechanical, thermal, and thermomechanical properties of polylactide (PLA) parts obtained by injection molding, were recently studied [107]. Due to a dual effect of plasticization in combination with molecular weight increase and branching reactions, it was found that PLA parts with CE in contents 2.5–7.5 wt% showed an increase to all mechanical properties as well as to thermal stability. In a recent study, polyaryl poly(methylene isocyanate) (PAPI) was used as a chain extender for a high molecular weight PLA material with better rheological, thermal and mechanical properties [108]. According to Khankrua et al., the addition of polycarbodiimide (PCD) at a ratio of 0.5 wt% can prevent the further mechanical degradation and thermal instability caused by high processing temperatures [109]. The GPC results showed that the molecular weight (MW) of processed PLA tends to decrease when increasing the processing temperatures (from 220 to 250 °C). Additionally, its molecular weight distribution (MWD) showed a bimodal distribution, which can be justified by the existence of polymer chains that did not react with the chain extender and those that reconnected by the chain extender and thus doubled in size.

Similar types of effective chain extenders were mentioned that could be block copolymers with other reactive groups. According to Hung et al., the addition of reactive block copolymers such as poly(styrene-b-methyl methacrylate-b-glycidyl methacrylate) (PS-b-PMMA-b-PGMA, PSMG) and poly(styrene-b-glycidyl methacrylate) (PS-b-PGMA, PSG) can increase the molecular weight and melt strength of PLA [110]. Furthermore, it was reported that the existence of MMA and styrene segments, can enhance miscibility with PLA and act as nucleating agents to promote crystallization. The addition happens to dried PLA in a twin-screw extruder at 180–200 °C at a ratio of 1 wt% (CE/PLA). The MMA segments are the key structure of the chain extender and the optimum ratio of styrene to MMA is at 1.0–1.2. The MW of processed PLA significantly increases (up to 4×) by adding PSMGs or PSG.

All the above-mentioned CE are low molecular weight copolymers. However, simple bifunctional molecules such as 1,3-phenylene-bis-2-oxazoline (PBO), pyromellitic dianhydride (PMDA) and 1,10-carbonyl bis caprolactam (CBC) (Scheme 11) have been also examined as appropriate CEs [111]. The additional stage and extenders’ concentration are key issues in the selection of the ideal conditions for the modification of PLA by chain extenders. According to this research, the most promising results were obtained by adding 1 wt% of PBO processing with melt polycondensation at 160 °C, 1 wt% of PMDA processing with oligomer polycondensation at 130 °C or with melt polycondensation at 160 °C and finally 0.25 wt% of CBC with oligomer polycondensation at 130 °C or with melt polycondensation at 160 °C too.

Core-shell modifiers with silicone copolymer as the core and methacrylate-co-glycidyl methacrylate (GM) copolymer (PASi-g-PMMA) as the shell, can be used to improve the toughness of PLA and thus to reduce its brittleness [112]. The GM groups played the double role of compatibilizer and chain extender, which not only improved the interfacial adhesion between the PLA and PASi-g-PMMA particles, but also increased the molecular weight and chain entanglement of PLA. Apart from the mechanical properties, chain extension has been proven to significantly improve the thermal stability of melt PLA, its rheological properties, while protecting it from hydrolytic reactions and depolymerization during its process [113]. To overcome the challenge of melt processing and degradation efficiency of PLA in the industry, the use of phosphites was studied by Sirishina et al. [114]. During melt extrusion, phosphites such as tris(nonylphenyl) phosphite (TNPP) and tris (2,4-di-tert-butylphenyl) phosphite (TDBP) function as a processing aid, besides acting as a chain extender for the stabilization of PLA’s molecular weight.

## 5. PLA Applications

**Figure 6:**
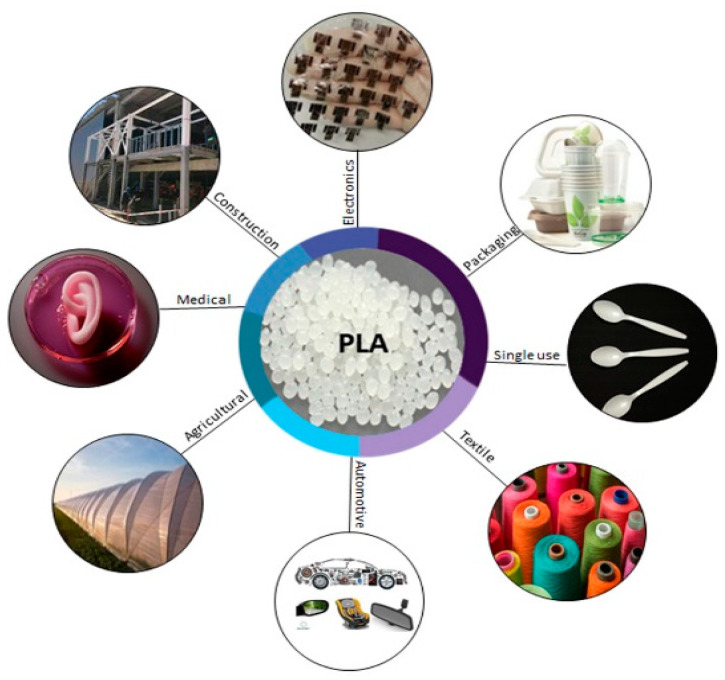
PLA applications in end-use industries and its’ global market consumption.

The global PLA market extent was priced at USD 525.47 million in 2020 and is estimated to expand at a consecutive annual growth rate (CAGR) of 18.1% from 2021 to 2028 [115]. The request for the product mostly originates from the end-use industries, such as biomedicine, automotive, construction, agriculture, single used items and food packaging, mostly for replacing petroleum based plastics (Figure 6) [116]. The global PLA market consists of six regions: Asia Pacific, Europe, Latin America, North America, Middle East and Africa. Asia Pacific is anticipated to be the fastest-growing regional market from 2021 to 2028, owing to constant development of the automotive as well as packaging industry. PLA demand is going to be increased a lot in countries of Asia Pacific such as Malaysia, China, India, Vietnam, Thailand, Singapore, South Korea, and Japan owing to high adoption rate by the automobile manufacturers. North American countries such as the U.S and Canada are estimated to witness a significant regional market growth in PLA. This can be explained based on consumers’ preferences in these countries for packaged foods, including frozen and ready-to-eat meals. In addition, it is anticipated that Mexico and Latin American countries will exhibit a remarkable growth in PLA market due to rising automotive production and electronic products respectively. Europe is the largest PLA market followed by North America and Asia Pacific. Poland, Russia, France, Spain, the UK, and Germany have emerged as the main market consumers of PLA due to the continuous development of their transportation sector. Moreover, Middle East and Africa are estimated to witness a substantial growth in the textile industry. Simultaneously, the fact that less carbon emissions are formed by PLA in comparison with conventional plastics is considerably boosting its demand across the globe [117].

The predicted global growth of PLA market lifted the competition among the leading companies in terms of LA and PLA production, the product quality and technology advancements used in the manufacturing process [118]. Corbion Purac is the leading producer of LA in global industrial scale, with a total of over 200,000 metric ton LA capacities. 140,000 tons of LA plant are located in Thailand (next to the PLA plant under Total Corbion joint venture) plus multiple plants around the globe. The second largest LA plant is Cargill’s in the USA (next to the NatureWorks PLA plant under the joint venture between PTT Public Company Limited and Cargill). Jindan in China is owner of a ~100,000 tons LA plant since the beginning of this century. In Belgium, Galactic owns a LA plant and is converting its 40,000 tons in China to 80,000 tons with BBCA (by 2020). Several other companies such as Golden Corn and Baisheng are constructing new LA plants by genetically engineered strains [9].

### 5.1. PLA for Packaging

One of the main PLA consumers is the packaging industry, primally in the sector of food packaging. The wide utilization of PLA in the manufacturing of bottles, jars, and containers, as well as in fresh food packaging solutions, was accounted for the largest revenue share of over 36% in 2020. Furthermore, the inclination towards sustainable packaging and growing ecological alarms throughout the world are compelling companies to use the product in numerous packaging solutions. Many countries, such as the U.K., Zimbabwe, New Zealand, Taiwan, and other states of the U.S. (such as Hawaii, New York, and California) implement a firm ban on single-use products, thus significantly forcing the product request in the packaging end-use sector [115]. PLA has been widely studied for use in food packaging because it has great biocompatibility, good physical properties including high strength, thermoplasticity, processability, and its non-toxicity. However, its low flexibility, poor crystallization behavior, and inefficient barrier properties are some of the disadvantages that restrain its application. Therefore, in order to increase its performance various substances are often mixed with appropriate additives or with other polymers that have higher gas barrier properties such as furanoates [119]. The potential of antimicrobial PLA applications in packaging applications has been studied [120]. Films with antimicrobial properties have been produced from biodegradable PLA and silver offering a sustainable solution for food packaging industry [121]. At a glance, Risyon et al., synthesized PLA/halloysite nanotubes bionanocomposite which exhibited great mechanical properties, water and oxygen permeability, thus indicating high potential for food packaging applications [122]. Antibacterial polylactic acid film incorporated with cinnamaldehyde inclusions for fruit packaging demonstrated excellent oxygen resistance, water resistance, and tensile strength [123]. Ghozali et al., investigated the effects of various metal oxides addition in PLA film for antimicrobial packaging applications [124]. Additional studies have been also mentioned in literature and their compositions and properties improvement are reported in Table 2.

**Figure 7:**
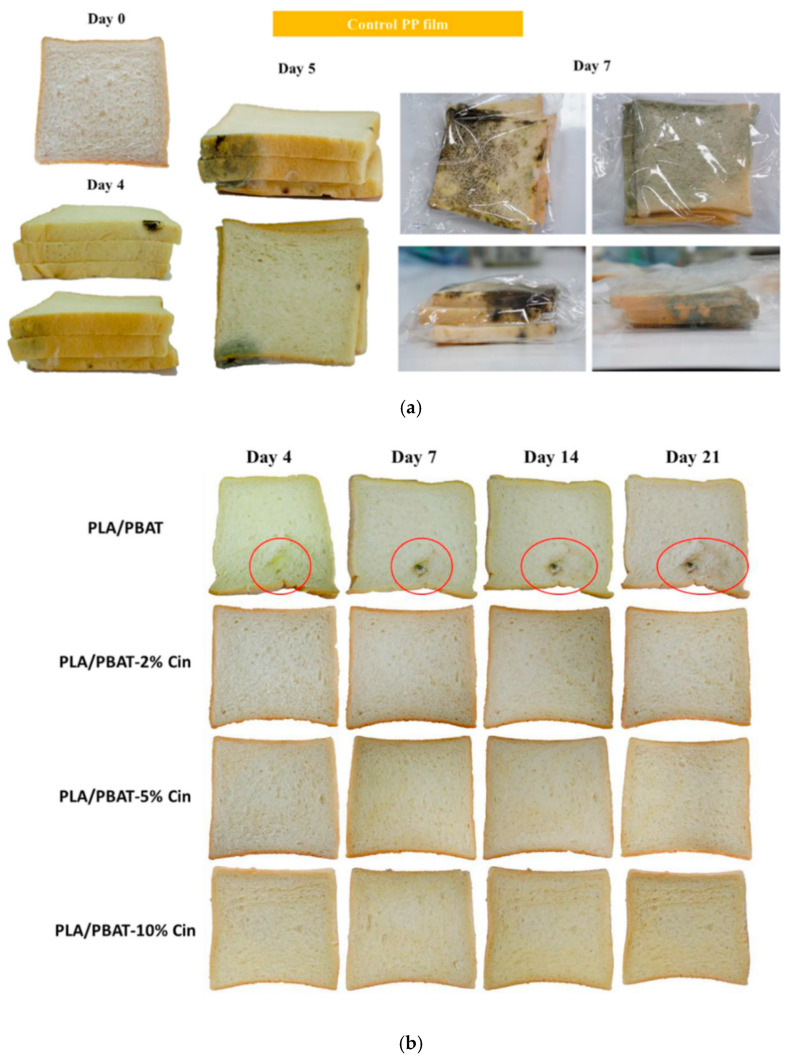
Appearance of packaged bread stored in (**a**) PP and (**b**) PLA and PBAT blend films with different concentrations of transcinnamaldehyde (2%, 5% and 10%) for 21 days at 25 °C. Circles indicate mold growth on bread Reprinted with permission from ref. [134]. Copyright. 2020 Elsevier Ltd.

Antifungal bioplastic films were developed based on PLA and poly(butylene adipate-co-terephthalate) (PBAT) blends with incorporated trans-cinnamaldehyde [134]. It was found that bread stored in conventional PP films exhibited mold signs on day 4 while further storage resulted to expansion of fungal growth that spread over the whole bread (Figure 7a). PLA/PBAT films effectively limited expansion of mold mycelium while microbial growth was not detected in all films containing trans-cinnamaldehyde (Figure 7b). This is due to high quantities of trans-cinnamaldehyde release that effectively inhibited the microorganisms.

**Figure 8:**
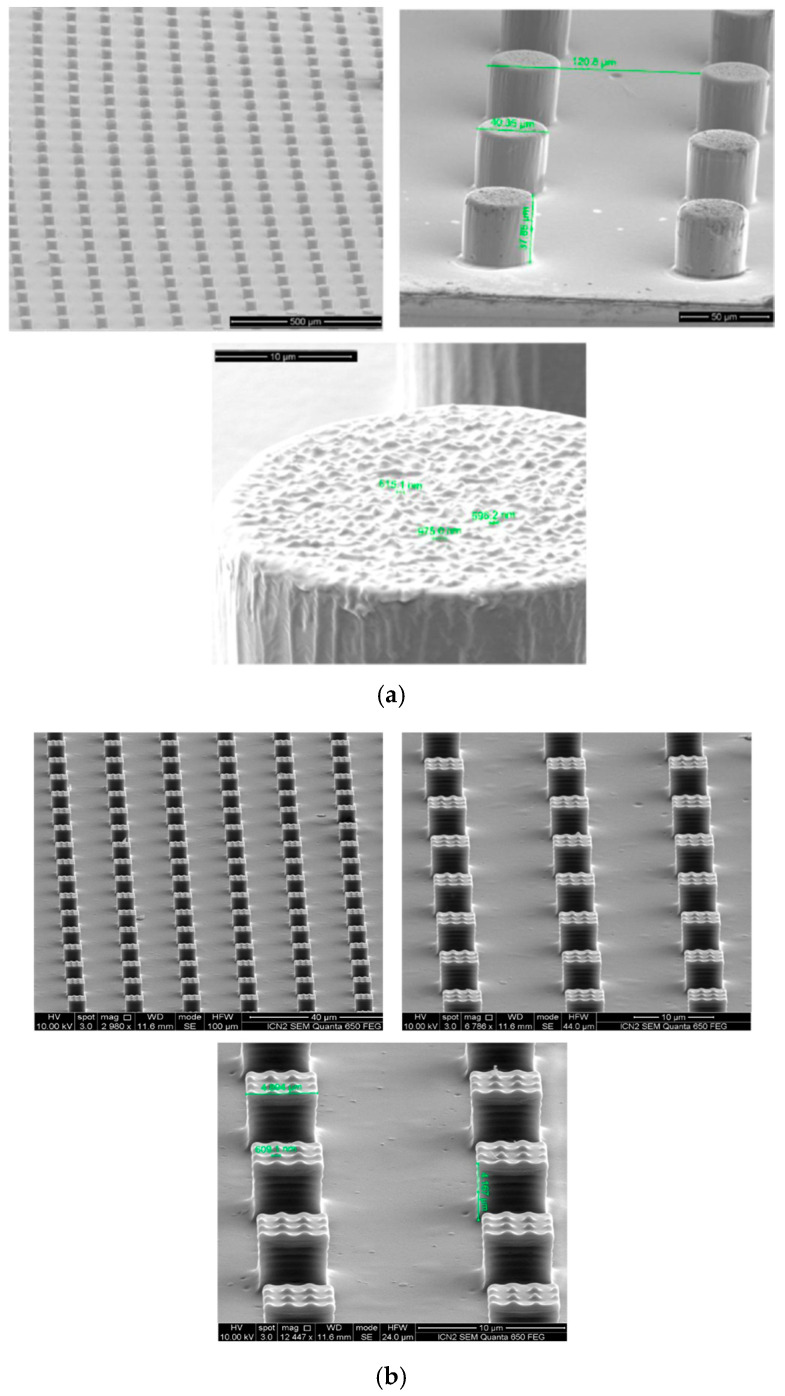
SEM micrographs of 3D hierarchical domains on the surface of PLA films at different magnifications: (**a**) black silicon nanostructures on spherical micropillars [136]; (**b**) rectangular hierarchical nanostructured PLA nanocomposite surfaces [137].

In our previous works it was proved that antimicrobial PLA films can be prepared when hierarchical nanostructures are patterning on film surfaces using nanoimprinted thermolithography [136,137]. The successful formation of the 3D hierarchical nanostructures on PLA films’ surface was proved by SEM (Figure 8). It can be seen, that micropillars with different widths and heights decorated with black silicon (Figure 8a) or cone-and needle-shaped nanostructures (Figure 8b) on micropillars in the area on nanometers.

**Figure 9:**
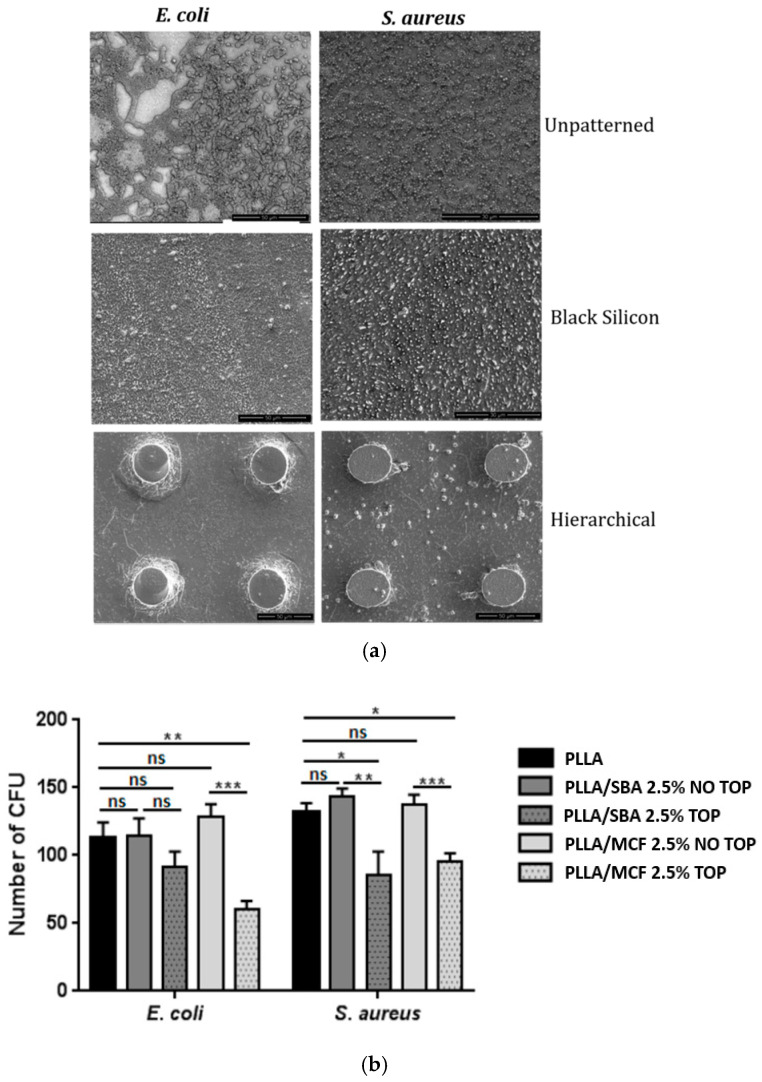
(**a**) SEM analysis, the reference strain SEM observations of “rod-shaped” *E. coli* and “coccoid-shaped” *S. aureus* on flat unpatterned, black silicon, and hierarchical domains on the surface of the PLA films [136]. (**b**) Number of CFU formed on the surface of the films prepared in this work [137].

The bactericidal behavior of the nanopatterned films were investigated by incubating Gram-negative *E. coli* and Gram-positive *S. aureus* on each film surface for periods up to 24 h and assessing their bacterial viability. As shown in Figure 9, lower cell densities for both bacteria were measured on all nanostructured surfaces, compared to the unpatterned films. Taking these results into consideration, it can be concluded that the nanocomposite PLLA films’ surfaces enhanced with micro/nano-topographical features inhibited significant bacterial growth compared to those with flat or unpatterned surfaces, as the available area for bacteria attachment is reduced.

### 5.2. Single Use Products

In the case of single use products, increasing efforts are being made to reach an autonomous society and economy based on non-renewable supplies. The use of greener materials deriving from PLA composites as commodity plastic alternatives, is such an example. The European Commission (EC) is actively involved on this matter, thus supporting various projects and activities, aside from defining strategic routes to follow.

A recent document revealed the necessity of enhancing the plastic recycling procedures in Europe, by reducing the landfilling areas and endorsing the plastics recycling improvement. The document mentioned above brings into question the use of oxo-degradable polymers, mostly due to its disintegration in marine environments and its undesirable consequences on marine fauna and flora. The objectives of the EC against plastic remnants are clear in its banning and/or restriction of single-use plastics (SUP), as declared in the Directive (EU) 2019/904, although sometimes may be misleading. Although the majority of the population is highly worried about the negative influence of plastics in the ecosystem, the customers are not only the ones to be held accounted for, but also the producers. This means that they must adapt their methods in order to deliver more environmentally friendly products and goods [138].

A numerous variety of single use products including lids, hot and cold cups, plates and bowls, cutlery sets, trays, straws and containers is commercially available in the recent years. Furthermore, fully biodegradable PLA gloves, bags of all kinds, gift boxes as well as animal wastes bags can also be traced in the market. In the field of research, great progress is being achieved in synthesizing novel products from PLA. Delgado-Aguilar et al., synthesized PLA blends with PCL, a topic which has gain increasing interest through years, by means of batch extrusion and processed in an injection molding equipment. The development of improved toughness materials remains one of the core objectives of PLA/PCL blends while sustaining the biocompatibility and biodegradability of both phases. Nevertheless, their mechanical performance at high temperature is one of the major limitations of PLA and PLA/PCL blends. In addition, low melting temperature of PCL limits their use in applications where extreme temperature is mandatory. However, the glass transition temperature of PLA often governs the softening temperature of products prepared from neat PLA or PLA/PCL blends. Melting PLA with PCL may reduce the brittleness of PLA, thus providing a variety of possible uses, particularly those related with toughness. However, reports indicate that the incorporation of PCL may also have an undesirable impact on tensile strength. The incorporation of PCL considerably increased the toughness of PLA, by obvious increase of the energy essential to fracture the samples. Overall, it was observed that the acquired PLA/PCL blends can constitute a firm and ecofriendly substitute to oil-based commodity materials [138].

Zhao et al., studied the effect of various sterilization methods on the properties of commercially accessible biodegradable polyesters such as PLA, PBAT and their blends. The samples were prepared by compression molding and were exposed to four sterilization treatments involving electron beam (EB), ethylene oxide gas (EtO) saturated steam (SS) and hydrogen peroxide gas plasma (HPGP). The findings of the study indicate that electron beam can be applied in PLA and its blends, while EtO and SS is not recommended due to shrinkage of samples and change from transparent to opaque. In addition, blends of PLA with PBAT cannot be treated with HPGP sterilization method. To conclude, this study exhibits that, when a suitable sterilization process is selected, PLA offers the possibility of being used for transparent medical devices such as the barrel of syringes or microfluidic chips. Furthermore, PLA/PBAT blends find application in non-transparent medical packaging [139].

### 5.3. PLA for Textiles

PLA is being considered as the most promising biodegradable fiber produced from renewable resources for replacing conventional PET fibers in textile industry. PLA fibers have properties such as PET fibers, but some of them differ. PLA brings together the ecological advantages along with the excellent performance in textiles and fills the gap between synthetic and natural fibers. On the other hand, unlike PET, PLA may be subjected to thermal degradation, and has poor hydrolytic resistance to strong alkaline. This can affect the parameter setting and method selection while producing and processing PLA fiber or fabrics. Fibers made of PLA find a wide range of uses, from pharmaceutical and medical applications to environmentally friendly films and fibers for packaging, clothing, and houseware [140,141].

PLA fibers have a significant application in packing industry but only around 2% of PLA is used in the form of textile fibers [142].

PLA fibers are primary applied in duvets, pillows, comforters, mattresses and can also be found in nonwoven applications such as wipes, hygiene products, agricultural and geo textiles. PLA fabrics are used in clothing for fabrication of activewear, underwear and fashion wear, since they exhibit good moisture management [140]. Its combination with other biobased or symbatic polymers, is very common in textiles industry, for the production of a reinforced fiber with enhanced properties. PLA can be used in blends with wool, lyocell, and cotton, or alone. Some examples of those combinations are presented in the following table (Table 3).

The use of PLA in textiles is expected to be increased in the near future due to its great ecological advantages as well as its good technical performance. In addition, major attention needs to be given as complicated wet processing, such as pre-treatments, dyeing, clearing, and subsequent finishing treatments, is required [140].

### 5.4. PLA for Automotive

PLA finds application in the engineering of parts used in the automotive industry, particularly as interior components and under-the-hood gears. Its extraordinary bio-content makes the product well known for decreasing the carbon footprint and provides various benefits, such as improved impact and UV resistance, high gloss, and dimensional stability. All the features mentioned above, constitute PLA as the perfect substitute to most of the conventional thermoplastics, such as polycarbonate (PC), poly (ethylene Terephthalate) (PET), acrylonitrile butadiene styrene (ABS), poly(butylene Terephthalate) (PBT), and polyamide which are traditionally used in automotive engine compartments and interiors [115].

PLA bio-fiber composites are globally utilized in a wide range of applications including automotive interiors. Biofibers or fillers are the easiest and most environmentally friendly materials to improve the mechanical and thermal properties of PLA. In Mercedes-Benz E-class there is a rich utilization of plant fiber. These bio-fiber based biopolymers are used to develop various automotive components, such as dashboards, door panels, package trays, headliners and some interior components [147]. A potential route in improving the performance of PLA composites appears to be the use of nanofillers and appropriate processing techniques. Graphene reinforced PLA nanocomposites overcome the major limitations of thermal stability and high moisture absorption behavior which are present in the automotive industry [148]. Additional PLA composites were reinforced with other fibers in order to achieve improved properties. Some examples are presented in the following table (Table 4).

### 5.5. Agricultural Uses of PLA

After decades of agriculture development, global food security is facing enormous threats. Soil degradation and pollution, water contamination, protection from crop pests as well as the upcoming upheaval caused by climate change, are major challenges to feed a growing human population with products which often end up in the environment as post-consumer waste [152,153,154].

The solution to the problem is to replace traditional products based on refined oil products with polymer materials which will be derived, from plant-based products such as corn or soybean. Currently, in agriculture packaging and textile industries, non-degradable plastics are being replaced with environmentally friendly polymers in order to reduce the amounts of solid waste, whose recycling may be impossible [154,155,156].

Hydrophobic surface treatments were applied to commercial PLA exclusion nets and FDM-printed meshes. Commercial PLA nets have been modified from superhydrophilic to hydrophobic after two surface treatments were applied: a novel solvent-induced treatment (DDD), and a chemical vapor deposition approach (PICVD). FDM printed sample hydrophobicity was increased by up to 96% to 143 using this treatment (DDD). Water access through the meshes was lowered by the hydrophobic surface treatments, providing that the netting was tilted at 60. Finally, the developed method of PLA surface structuration and its application for increased molecule adsorption is promising in the field of future development of environmentally friendly control methods [154].

Spun-bonded nonwovens made of PLA were studied for the physiochemical properties and their biodegradability. Agricultural nonwoven PLA fibers were completely decomposed in simulated laboratory composting during 16 weeks of incubation. The total sensitivity to degradation in compost is also confirmed by observation of changes in appearance and the structure of the samples using SEM [157].

Polylactic acid/polybutylene adipate terephthalate (PLA-PBAT) mulch was tested in two different soils for their antimicrobial properties. The study of the results for seven months, showed that PBAT-PLA mulch on a cotton crop changed both soil bacteria abundance and the specific species distribution [158]. Biodegradable plastic mulches show great properties. They are remaining stable under convenient or dry storage conditions, in low temperature, in the dark and in indoors storage. PBAT-PLA film was tested for integrity and its properties have been proved stable after one and two years of storage [159,160]. Flexible films are intensely and increasingly used in agriculture in the last 15 years. The environmental impacts increased due to their incorrect post-use waste management, thus gradually rendering degradable films a valuable alternative. PBAT/PLA composites, reinforced with calcium carbonate CaCO_3_ particles, were produced by melt extrusion using different contents of CaCO_3_ leading to inexpensive and flexible films [161].

In another work, two light conversion agents were prepared in a solution by combination of europium with organic ligands. Two types of biodegradable conversion mulch films, Eu(DBM)_4_CPC and Eu(TTA)_3_(TPPO)_2_ were successfully prepared under industrial blown film conditions, using polylactide and poly(butylene adipate-co-terephthalate). Synthesized films showed excellent light conversion ability and high color purity, while earth complexes improved their melt flowing property and decreased the melt viscosity of the blend. In addition, the elongation at break of the film highly increased. The rare earth complexes can induce main chain of PLA scission, causing rapid molecular weight reduction, while DSC results indicate that both rare earth complexes can improve the crystallization of PLA [162].

### 5.6. PLA for Electronic Devices

**Figure 10:**
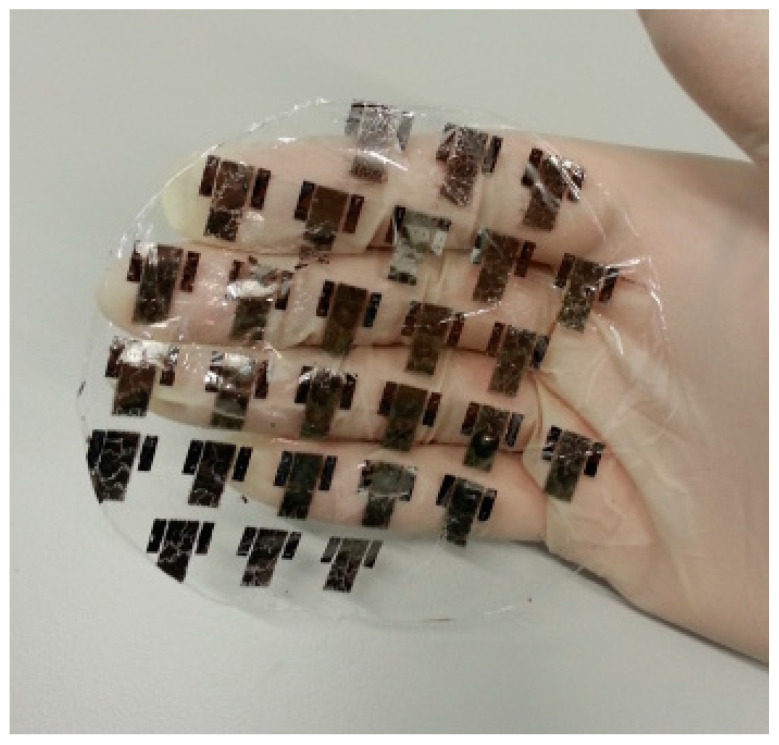
Array of organic field-effect transistors fabricated on PLA Reprinted with permission from ref. [165]. Copyright 2014 Elsevier B.V.

PLA is also extensively used in the electronic industry due to its unique characteristics [21]. Electronic devices and personal electronic products (TVs, smartphones, wires, cables, gaming systems, portable computing), appear to have the highest growth rate in the electronics sector (Figure 10). China is the largest base for electronics production in the world, due to the domestic demand for electronics, but also the exportation of electronic products to other countries. The fabrication of electronic products from renewable PLA material can obtain energy savings in small electronic applications and their energy storage and reduce the depletion of limited material resources [163]. Hence, while the demand for PLA in China is already great, it is reported to grow even more in the next decade [164]. Several studies have mentioned the preparation of PLA based products in electronic devices. An example is the fabrication of spin-coated biodegradable PLA thin films. The full mechanical and electrical characterization of these films showed that they exhibit great mechanical and dielectric properties and make them suitable for the fabrication of disposable electronics [165]. Furthermore, bio-based PLA and recycled polyethylene terephthalate were combined in order to be used as a substrate in organic photovoltaics. Ultra-thin biofilms decreased the carbon footprint, about 10–85%, the energy payback times from ca. 250 days to under 10 days in outdoor light and less than 1 year in indoor energy [164]. However, PLA exhibits poor heat resistance and inherent brittleness. Enni Luoma et al. in an attempt to overcome these drawbacks, increased the PLA film crystallinity through orientation and annealing for printed flexible and hybrid electronics. They used two commercial grades, standard PLA and a high heat PLA, plus one stereocomplex PLA so that to compare PLA performance with different optical purities and crystallinity. In high heat PLA the crystallinity increased from 0% to 50% improving tensile strength by 83%, strain at break from 3.7% to 114% and tensile modulus by 52%. The stereocomplex PLA provided higher temperature stability. The final melting was observed at 220 °C while its optical transparency reached 95%, remaining high to 250 nm wavelength [166].

### 5.7. PLA for Construction

**Figure 11:**
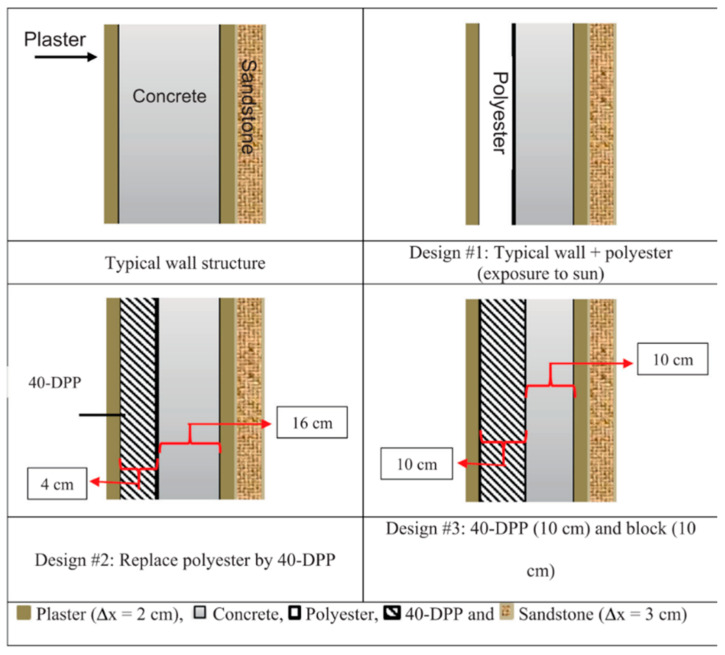
Components and designs of a wall structure with PLA and date pit powder Reprinted with permission from ref. [168]. Copyright 2020 Elsevier Ltd.

The construction industry is one of the fields that consumes significant global resources which unfortunately cause negative impacts to the surrounding environment. Consequently, increasing efforts have been made to establish a sustainable building design in order to minimize the severe impacts induced from construction [167]. During the last few years, PLA is gaining prominence as alternative material for construction sectors. PLA composites are renewable in nature, economical, biodegradable, and compatible with most of the manufacturing processes for structural applications. Because of their thermal insulation, green blends of PLA/date pits powder find application in building construction, thus improving the thermal management with respect to buildings and allowing the partial replacement of concrete used in a typical building wall (Figure 11) [168]. Epoxy coated PLA is also targeting to replace river sand in concrete in walls [169]. Yarns made out of the stem of the most widely cultivated fruit in Sri Lanka, banana (469,842 tons per year, 49,168 hectares), were used as a reinforcement and PLA was used as the matrix in order to synthesize composites for partitioning walls [170].

### 5.8. Biomedical Applications

#### 5.8.1. PLA in Bone Tissue Engineering

One of the largest medical applications of PLA is the preparation of scaffolds in tissue engineering. Tissue engineering is the most recent innovative domain where these biodegradable materials provide surfaces that promote the regeneration and reconstruction of human tissue and organs. PLA presents great physical, chemical, biomechanical and degradation properties which make it a suitable material for bone, cartilage, ligaments, skin, blood vessels, nerves, and muscles fixation [171]. In recent years, the technique of 3D printing has been widely used in web engineering. The 3D printing technique is based on a controlled dispensing system integrated with pumping technology and a CAD/CAM system allows the precise and reproducible fabrication of 3D structures with well-defined predetermined geometries 3D printed PLA-based scaffolds. PLA emergence in 3D printing technology led to development of scaffolds in desired shapes and forms, in low cost, with corresponding mechanical and biological properties and controlled micro/nano structures [172,173].

Nevertheless, PLA is usually combined with natural polymers to avoid possible toxicity and undesired inflammatory responses [174,175]. Several surface roughening, surface peptide modification, hyaluronic acid, and collagen surface modification, etc., have been studied in order to improve its poor biological activity [174,176]. PLA/hydroxyapatite (HA) scaffold filament was fabricated with HME and printed with FFF 3D printer, producing a printed scaffold with equally sufficient or even better precision of pure PLA filament printed scaffolds [177]. In a similar work, 3D-printed scaffolds of PLA/HA were loaded with enhanced bone marrow and induced membrane to repair large radial defects. The in vivo results showed that it can be used as an alternative to cure large bone defects [178]. Another 3D-printed PLA scaffold was immersed into polydopamine (PDA) and collagen type I solutions. Its activity in the cell culture/adhesion, and also the metabolic activity of porcine bone in the scaffold, revealed the improvement of biological properties of the scaffold after the PDA/collagen type I coating [179,180].

Bone flaws mainly result from trauma, infection, osteoarthritis, osteoporosis, malignancy, cancer, or congenital malformations. In clinical research today, research and development of bone replacement materials (bone tissue engineering) aiming to bone repair, are prevalent [181,182,183]. Scaffolds, which are used in bone tissue engineering, must have high porosity to transport cells to injuries and enable them to develop the formulation of new tissue [184,185]. PLA has been widely used to fabricate porous scaffolds for bone regeneration, utilizing different techniques, in combination with other synthetic and natural polymers, as well as bioactive ingredients [178,186,187,188]. PLA scaffolds loaded with polyethyleneimine (PEI) and citric acid (CA) derived products, were modified with calcium phosphate mineral deposits to create a bone environment. The mineral deposition of synthetic obtained PLA-PEI-CA/HA was nearly twice as high in comparison with neat PLA, and human mesenchymal stem cells adhesion and proliferation was ~50% higher [174,189]. PLA was combined with dicalcium phosphate dihydrate and hydraulic calcium silicate (PLA/DCPD/CaSi), fabricating a scaffold which was biocompatible and bioactive [190,191,192]. PLA, and halloysite nanotubes (HNTs) loaded with zinc nanoparticles (PLA+H+Zn) synthesized a biomaterial with great antibacterial and osteogenic properties for bone regeneration [193].

**Figure 12:**
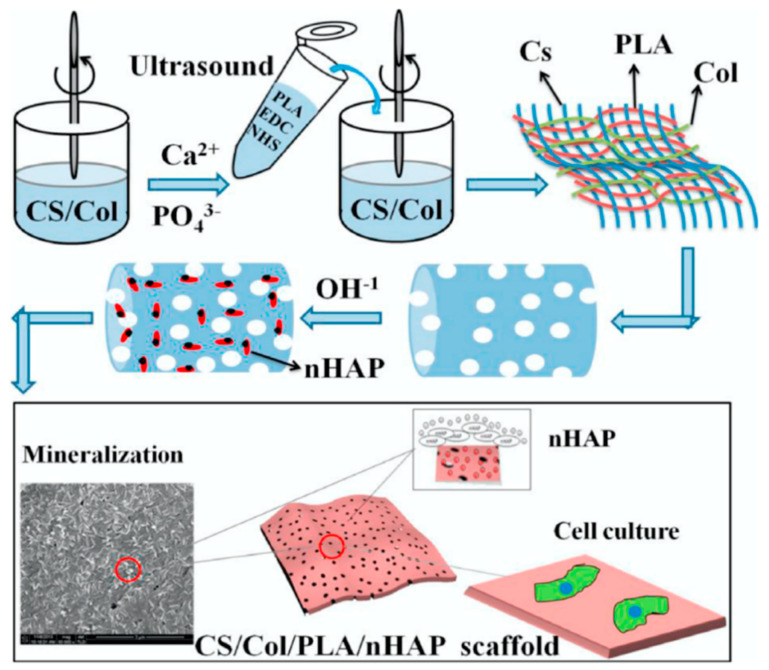
Schematic formation of the CS/Col/PLA/nHAP hybrid scaffold Reprinted with permission from ref. [194]. 2019 Elsevier B.V.

Additionally, PLA/Col/nano-HA/Chitosan (PLA/Col/nHA/CS) composite scaffold was fabricated. It was biocompatible, maintaining cell growth, appearing to have similar micro-nano morphologies to natural ECM (Figure 12) [194,195]. A novel PLA/PCL/HA scaffold was prepared by coating cuttlefish bone aragonitic structure with a PLA/PCL film, which combines the brittleness and degradation of PLA with the ductility of PCL. In another study where the prepared scaffolds were bioactive, they boosted cell attachment and proliferation, and supported calcium phosphate deposition [138,139,191,192,196,197,198].

In a previous work of our group, the crystallization behavior and in vitro–in vivo hydrolysis of PLA absorbable reinforcement ligaments, appropriate for orthopaedic applications and mainly for the repair and reinforcement of articulation instabilities, was studied [199]. It was found that the MW during in vitro hydrolysis at 50 °C has an initial slow reduction but after 30–40 days the decrease rate becomes much faster. Furthermore, as found from in vivo hydrolysis in the human body, the PLA reinforcement ligament is fully biocompatible and after 6 months of implantation is completely covered with flesh. However, the observed hydrolysis rate from in vivo studies was also too slow and this was attributed the high MW and degree of crystallinity [200].

#### 5.8.2. PLA for Blood Vessels and Organs

In addition to 3D printed scaffolds and tissue engineering for bone regeneration, PLA has also been used in the treatment of blood vessels and organs, as well as for the fabrication of dental implants [201]. PLA was combined with metals to produce biodegradable materials for next-generation coronary stents and biomedical devices. Specifically, Fe was coated with PLA, and the obtained composite was implanted into the porcine artery in order to accelerate the corrosion of iron. The Fe/PLA stent exhibited excellent functionality in the interventional treatments of cardiovascular diseases [202]. A PLA/PCL foam vascular scaffold was prepared with supercritical CO_2_ and was etched off with NaOH, to open pores and to improve its hydrophilicity. The scaffold showcased excellent fracture resistance and suture retention [203,204]. Vascular grafts were fabricated using PLA with FMD (fused decomposition modelling) 3D printing method. The scaffolds that were created can be applied as next generation vascular grafts with adjustable porosity and pore size [147]. By altering the flow rate of polymer, different porosities and pore sizes were fabricated from a solid CAD model. The results indicate that porous structures with smaller pores the resolution of FDM are possible. In vitro degradation and biocompatibility of 3D printed grafts were investigated, with results indicating that PLA grafts met the requirements of tissue engineering. The degradation study of PLA revealed that it will degrade slowly and provide enough mechanical support in vivo for cell growth [147].

#### 5.8.3. PLA for Skin Regeneration

The human body is mainly consisted of skin, the largest organ and the first defensive mechanism of our organism. When the functions of the skin are disrupted, physiochemical procedures take place in order to fix the wounds. However, chronic wounds do not heal through the usual process [205,206,207]. PLA has contributed extensively to the composition of scaffolds for wound healing and tissue repair [205,207]. PLA matrix nanofibers encapsulating doxycycline (DCH) metalloproteinases inhibitor was fabricated by electrospinning for the treatment of chronic wounds. A study on diabetic rats showed advancement of the chronic wound healing, and greater efficiency prosses over topical coating of DCH because of the sustained release of DCH [208]. It was proved that the embedding of silver antioxidant nanoparticles (AgNPs) in PLA/PEG structure led to the fabrication of a skin dressing with simultaneous antimicrobial, antioxidant, and wound healing properties [206]. Scaffolds with a PGA core and PLA shell by coaxial electrospinning presented incredible abilities in wound healing. The scaffold was biocompatible, hydrophobic, appeared beneficial to cell proliferation and wound healing, presenting more than 90% re-epithelialization [209,210].

### 5.9. Medical Applications

#### 5.9.1. PLA for Tumor-Targeting

The most effective and conventional therapy against tumors is chemotherapy. Polymeric micelles have also been widely studied for targeting delivery of cytostatic drugs in order to limit chemotherapy’s side effects such as drug abuse due to wrong evaluation on real time therapeutic effect, tumor heterogeneity both the same tumor or in different tumors, low bioavailability and side effects resulting from nonspecific drug distribution and chemo-resistance [211,212]. PLA micelles are stable without special functional groups and have great promising applications for targeting drug delivery. PLA stereo complex interaction-assisted polymeric micelles have excellent biocompatibility, improved stability, and reduced degradation rate, compared to other micelles [212,213]. Liu et al., tried to fabricate microspheres based on PLA so as to have the potential to work as vehicles for cells and carry them to designated locations in a magnetic field. PLA and PLLA microspheres were synthesized and studied, proving that PLLA microspheres lack cohesive force comparing to PLA. For this reason, PLLA was modified by super-paramagnetic Fe_2_O_3_ nanoparticles assisted with oxidative dopamine polymerization for cancer treatment field [204] poly(oxazoline)-b-poly(L-lactide) encapsulated with doxorubicin formulation equipped with peptide-18 (a peptide which recognize the breast cancer cells). The tracking of the cancer cells and their extermination was extensively studied. These findings suggest that the success of formulation promises a targeted therapy system for breast cancer [214].

#### 5.9.2. PLA for Drug Delivery

Drug delivery is the technology applied to introduce the drug to the desired body site for drug release and absorption, or the subsequent transportation of the active ingredients across the biological membranes to the site of action. The challenges for a drug delivery system includes the improvement of drug efficiency and safety by controlling the rate, time and place of release of drugs in the body. Over the past few decades, nano- and micro-particles are used extensively in drug delivery systems because they can be injected or deposited directly at the site of action, providing a very high local drug level over an extended period.

PLA-based particles are commonly used as controlled drug delivery systems of therapeutic molecules. However, they have several challenges to overcome, such as a low drug loading capacity, low encapsulation efficiency, and final sterilization. In an effort to overcome such obstacles, innovative formulations of PLA-based particles are being synthesized [201,215,216,217,218]. Microspheres of PLGA and PLA, were prepared with different techniques to improve regulated drug release of particles [219]. Galactosamine-modified poly(ethylene glycol)-poly(lactide) (Gal-PEG-PLA) polymers were synthesized and PEG-PLA-TPGS micelles were made to promote the oral delivery of curcumin, a hydrophobic drug. The results showed high sustained release properties, making it a candidate for targeted delivery to the liver [220]. PLA contributed to synthesizing a multi drug delivery system in lens capsule bag. A temperature-sensitive drug delivery system carrying dexamethasone, moxifloxacin and genistein nanostructured lipid carrier (GenNLC) modified by mPEG-PLA based on F127/F68 as hydrogel was fabricated and its properties were studied thoroughly. The results in situ showed a decrease in postsurgical inflammation, to avoid infection and reduce the incidence of secondary cataract while having a programmed release of quantity and release profiles to cut down the need of eye drops in order to prevent inflammation or infection and to decrease the posterior capsular opacification following cataract surgery [221]. PLA, nHA and Eudragit produced double-layer microspheres were loaded with human insulin-like growth factor 1, and the results provide a promising therapeutic suggestion in order to delay osteoporosis and articular degeneration [172,222]. Rhodamine B as a model drug substance was loaded into PLA microchamber arrays that were prepared by dip-coating a PDMS stamp into PLA. Rhodamine B was released within 13 days in a phosphate buffer solution. The PLA microchamber arrays can be applied as biodegradable drug depot system for the cover of implantable endovascular stent [223]. PLA/vancomycin-loaded noisome was designed through layer-by-layer nanocoating. The study shows that this coating is valuable for medical or orthopedic implants and medical devices that are susceptible to bacterial infections [224].

**Figure 13:**
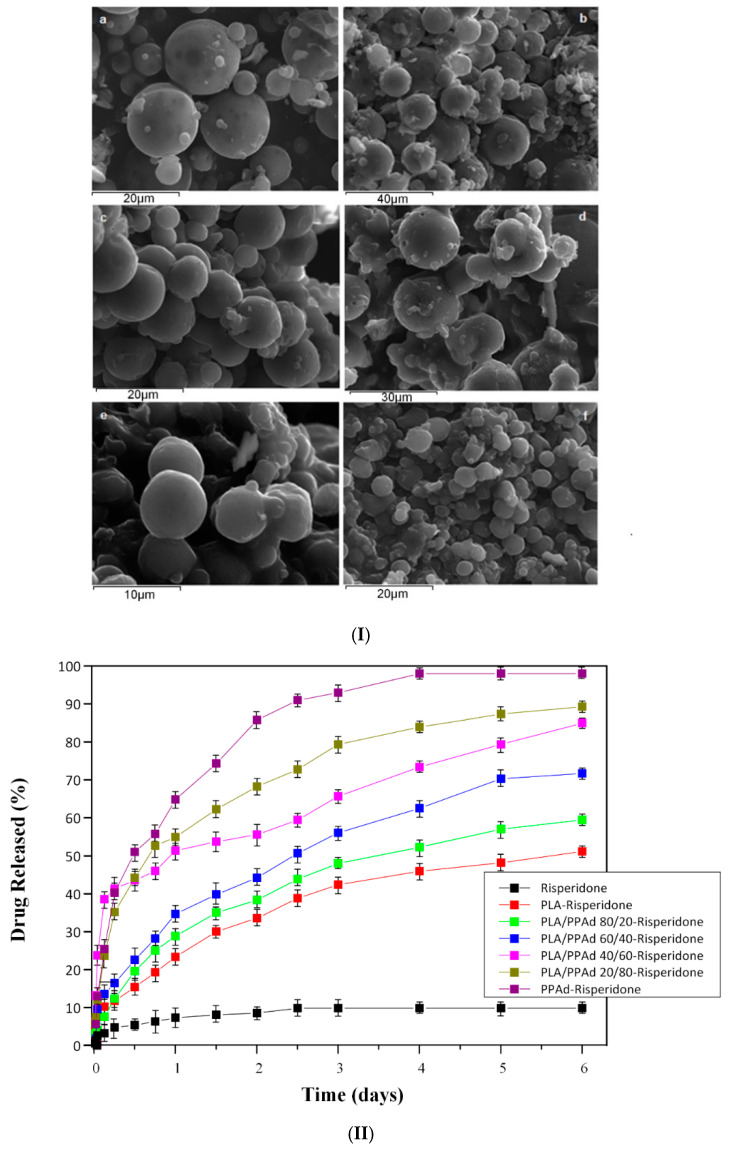
(**I**) SEM micrographs of risperidone microspheres prepared by neat PLA (a), PLA/PPAd 80/20 w/w (b), PLA/PPAd 60/40 w/w (c), PLA/PPAd 40/60 w/w (d), PLA/PPAd 20/80 w/w (e) and neat PPAd (f). (**II**) In vitro release profiles of risperidone drug loaded microspheres prepared using PLA, PPAd and PLA/PPAd blends [229].

Neat PLA or in the form of copolymers and nanocomposites has been used for several drug microencapsulations in order to prepare formulations with controlled release behavior, appropriate for long acting injectables [225,226,227,228]. In a recent study biocompatible PLA and PLA/poly(propylene adipate) (PLA/PPAd) blends have been used to prepare risperidone controlled release microspheres as appropriate long-acting injectable formulations [229]. Risperidone is a second-generation antipsychotic drug and a combination of low dose drug with long acting and extended release treatment is needed, in order to control the psychotic symptoms and treat schizophrenia. Figure 13a shows SEM micrographs of the prepared risperidone microparticles using PLA, PPAd and their copolymers. In neat polyesters such as PLA and PPAd, microparticles with spherical morphology and sizes varying from 2.5 to 15 μm and 1 to 5 μm have been prepared, respectively. In all studied copolymers similar microspheres with diameter size ranged between 2–17 μm have been also prepared, without big differences among the different polymer ratios.

The drug risperidone is a poorly water-soluble drug and its dissolution does not exceed 10% after six (6) days of testing (Figure 13b). When it was encapsulated into microspheres its solubility was substantially enhanced, which can be attributed to the amorphous or molecular level dispersion of risperidone inside the polymer matrix. Its dissolution profile is directly dependent from the used polymer matrix. The highest release was from PPAd microspheres (up to 95%) and the lowest from PLA microspheres (up to 40%) for a release time of six days. PLA/PPAd blends have dissolution profiles which ranged between the two neat polymers and by increasing PPAd amount in PLA/PPAd blends, the risperidone release rate increases too. This was probably due to the low melting point and glass transition temperatures of PPAd, compared to PLA, which both, as was found in a previous work, play an important role in drug release rate [230].

PLA has been used in the fabrication of scaffold matrices for drug delivery achievement with high loading and efficiency to specific sites. Different forms of PLA polymeric scaffolds for protein/drug delivery are available: (1) a typical three-dimensional porous matrix, (2) a nanofibrous matrix and (3) a thermosensitive sol-gel transition hydrogel. PVA/PLA core-shell structures were also obtained via coaxial electrospinning after altering the parameters that influence fiber morphology, such as external and internal flows, and voltage in order to correlate fiber properties with protein releasing abilities. The burst effect of coaxial electrospinning was eliminated. The optimal fibers demonstrated controlled protein release with the presence of shell and are considered a great candidate for bone guided regeneration [195,231]. Prefabricated electrospun PLA/HA/gelatin was used to create a scaffold, was immobilized with BMP-2 peptides through a PDA assisted coating method. A sustained release scaffold was produced, that synergistically promoted osteogenic diversity of bone mesenchymal stem cells. The scaffold had both in vitro and in vivo equally attractive biocompatibility and osteoinductivity [195,231]. In another work, PLA was 3D printed to create a scaffold combined with collagen, minocycline (MH) and citrate-HA (cHA) which showed high compressive strength and adequate wettability. The citrate-HA stimulated the adhesion, proliferation, and differentiation of precursor stromal populations. The 3D printed scaffold presented a satisfying antibiotic release profile, leading to local drug delivery therapy with antibacterial activities against *S. aureus* [232,233]. PLA also combined with chitosan, producing a bilayer scaffold by electro-spinning. NaX/Fe_3_O_4_ and Doxorubicin (DOX) anticancer drugs were incorporated into the scaffold and the drug loading rate of DOX was higher than 90%. After 7 days in the magnetic field, the DOX loaded PLA/chitosan/NaX/Fe_3_O_4_ managed to kill 82% of H1355 cells [195,234].

## 6. Technical Challenges, Limitations and Future Perspectives for Sustainable PLA Production

PLA is an eco-friendly polymer, and its production has many benefits compared with traditional polymers. More specifically, 65% less energy is required and also 68% fewer greenhouse gases are generated during its production procedure. Furthermore, it is a highly recyclable thermoplastic and contains no toxins. However, has also negative points. One of the biggest negative drawbacks is that lactic acid is derived from corn, one of the main food products. For this reason, new technologies should be applied in order to produce lactic acid by using glucose derived from plant biomass. Furthermore, PLA is a biodegradable polyester, but only under specific conditions that exist in industrial composting in a rich oxygen environment with high temperatures (58–80 °C), high humidity (>60% moisture) and in the presence of micro-organisms (thermophilic bacteria) [235,236]. Under these conditions, PLA could be degraded for more than 90% in short time ranging from 30–150 days and is converted by microorganisms into CO_2_, H_2_O and compost ingredients [237,238].

In the environment, at least 80 years [239] or 100 to 1000 years are required [240] for PLA’s decomposition, which means that PLA products should not be thrown into nature but only in industrial composters [237]. This is because PLA, as all polyesters, breaks down under certain environmental conditions via natural biological processes, such as the action of micro-organisms (mainly bacteria and fungi). The biodegradation mechanism includes enzymatic degradation, hydrolysis and the combination of them, and is a surface or bulk erosion procedure. Enzymes act as biocatalysts. However, hydrolysis of PLA in the environment only occurs in the presence of specific enzymes, such as Proteinase K. Thus, its hydrolysis rate is too slow because these enzymes are rarely ever found in nature. Soil burial experiments indicate that the degradation of PLA materials requires a great amount of time before starting, and the degradation rate is quite slow [241,242]. It was reported that even after one year under soil burial at 25 and 37 °C, no significant loss of mass was detected [243]. In a recent work the biodegradation of PLA films in soil matrix under mesophilic conditions was studied and it was found that the rate of mineralization is very slow (about 10% after 150 days) while there was no evidence of abiotic degradation of the polymer at 30 °C [244]. These studies are in total agreement with previous work from Briassoulis et al. group, where the soil burial tests for 7 and 11 months in soil environment under Mediterranean real field conditions. The tests showed that the degradation of PLA was slow and that it takes a long time for the material disintegration to start, even though degradation of the mechanical properties appears to start earlier [245,246]. The situation becomes even worse in marine environments due to the absence of any enzymes that could decompose PLA and due to the low temperatures, which rarely reach 30 °C, and thus PLA is unlikely to be hydrolyzed and remains stable for many years [247].

### 6.1. Technical Challenges

There are many technical and economical challenges that must be overcome in PLA production. First of all, the chemical synthesis method uses petroleum-based compounds which may subject to the possible supply problem of crude oil and its dramatic price variation. A limitation of this process is that it only produces the racemic (50:50) mixture of L-LA and D-LA (i.e., L/D-LA), which is not preferable for the drink, food, and pharmaceutical industry due to the metabolic issues that D-LA may induce. Furthermore, the PLA industry usually requires lactic acid with a high optical purity (e.g., ~99% L-LA and ~1% D-LA) [9]. In addition, during fermentation alkalis such as lime (calcium hydroxide solution) must be added to neutralize the lactic acid produced by microbes and control the pH of fermentation broth at an optimal level for the microbes. Therefore, formed calcium lactate salt must be acidified later by strong acids such as sulfuric acid to release lactic acid from the salt for further retrieval. However, this process typically generates gypsum (calcium sulfate), a disposable solid waste with limited use. After fermentation, lactic acid is retrieved from the broth and purified to meet its ending specifications. Depending on the raw resources, types of microorganisms, and media used, the recovering conditions and steps may need to be modified. A typical downstream process includes: (1) removal of biomass produced by microorganisms, (2) the above-mentioned sulfuric acid treatment, (3) removal of gypsum to recover crude LA, and (4) purification of LA by ion exchange chromatographic resins to manufacture food-grade LA enclosing some impurities, and/or by distillation to produce polymer (or heat-stable) grade lactic acid free of any sugar, protein, and most other impurities.

Crude lactic acid with impurities firmly affects the production procedure, the yield, and the properties of PLA. Therefore, the purification process of lactic acid monomer from any remaining impurities after the industrial fermentation is extremely vital. Furthermore, optical purity (in addition to the chemical purity) of lactide or lactic acid also has a great impact on the characteristics of poly (lactic acid).

Recently, genetically engineered *E. coli* strains have been used to obtain optically pure lactic acid with fast growth, high yield and conversion rates using defined media to further decrease the expenses [9].

### 6.2. Limitations for Commercial Applications

While PLA can be viewed as an eco-friendly biomaterial with outstanding properties, it also has many obvious negatives when challenged with requirements for certain applications: (1) PLA is very brittle, with less than 10% elongation at break, thus it is not suitable for demanding mechanical performance applications unless it is suitably modified. (2) Its degradation rate through hydrolysis of the backbone ester groups is too slow. (3) PLA is highly hydrophobic and can elicit an inflammatory reaction from the tissues of hosts [248].

Moreover, PLA degrades rapidly in months only at a high temperature (around 58 °C) typically under the industrial composting conditions, has poor water barrier properties and low heat distortion temperature when compared with traditional plastics. For flexible film applications such as mulch films or shopping bags, PLA needs to be blended with soft bio-degradable plastics such as poly (butylene adipate-co-terephthalate) (PBAT), typically in a 20:80 ratio in order for its flexibility to be enhanced. In other applications, PLA may demand some improvements and modifications due to its unique performance profile. After its combination with other compounds to improve its impact strength and heat stability, it may be used for engineering plastic applications such as the mobile phones, cases for computers, and radios.

PLA has an oxygen permeability of 2 mil/(100 in. day atm) and water vapor permeability of 18–22 g of 38–42 cc mil/(100 in.^2^ day atm), CO_2_ permeability of 183–200 cc mil/(100 in.^2^ day). This high gas permeability corresponds to a poor barrier property, a fact that restrains PLA from being used in some beverage bottle applications. Improvements are required for enhancing the barrier properties of PLA, potentially by combining it with high barrier plastics, for example poly (glycolic acid) (PGA), through lamination or melt compounding. Taking into consideration all the disadvantages of PLA mentioned above and its high cost, it is not surprising that PLA has not gained the attention it deserves in the previous years. In the past years, outstanding efforts have been made towards the enhancement of PLA performance via different modification routes [9,249].

### 6.3. Future Perspectives

According to the nova-Institute, the international bioplastics market is expected to significantly grow by 2020. At present, 25 companies are producing PLA worldwide, and the current production capacity reaches over 180,000 tons. The largest industrial-scale producer, Nature Works (Minnetonka, MN, USA), will build a new factory in Thailand in an effort to double the production capacity (now 140,000 tons/year) [250]. Almost certainly, Nature Work will build another 70,000–150,000-tons plant to meet the market requirements of around 500,000 tons by 2021–2022 [9].

The other producers, with a current production capacity of 1500–10,000 tons/year, are also planning to increase their facilities. Summation of these future capacities reaches 700,000 tons/year by 2020 [250]. Hisun built a 5000-tons PLA line in China then added a 10,000 tons line in 2017. Several new enthusiastic PLA projects were announced in China recently but may not actually be implemented. Nevertheless, in 2018 in China a 20,000 tons line was set up. Hengtian built multiple lactide-to-PLA fiber lines with an overall capacity of 10,000 tons while COFCO installed a 10,000 tons lactide-to-PLA plant. A 10,000 tons lactide plant is also being constructed by Jindan to provide lactide to other PLA plants who can only use lactide instead of lactic acid as the raw compound to polymerize PLA. Moreover, Synbra built a 5000 tons line to fabricate Expandable PLA (BioFoamTM) to substitute EPS- based foamed products. Other PLA facilities installed up to the present time are smaller pilot plants for analyzing the technology and feasibility of the process. Numerous new projects have been announced, such as BBCA-Galactic’s ~40,000 tons line and Hisun’s new 30,000 tons line (on the top of its 15,000-tons plant) to be fulfilled in 2020. Should these projects be successful, PLA supply capacity in the world will increase from 275,000 tons to 375,000 tons by 2020. Table 5 summarizes the present industrial PLA projects with the overall production capacity over 300,000 tons by 2020 [9]. The engagement of many new corporations, scale up of the existing companies’ facilities, globalization of the products and interest of international environmental agencies and governments may help to replenish the non-degradable plastic products almost completely in near future [251].

**Figure 14:**
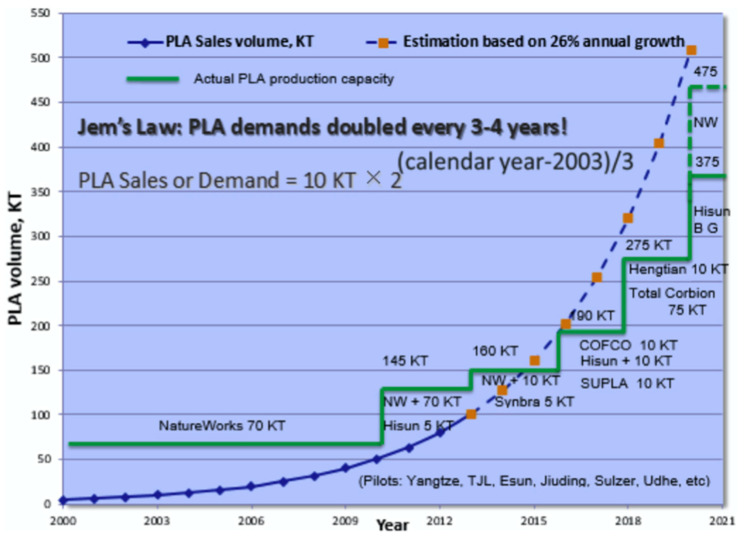
The Global PLA Plant Capacity vs. the Market Demand Predicted by Jem’s Law [9].

Furthermore, it is estimated by Jem’s Law that the global PLA market requirement doubles every 3 to 4 years (Figure 14). To conclude, with the advances of production technology and government regulations on plastics, PLA is expected to be extensively developed for non-medical applications [9].

## 7. Conclusions

In the last two decades, PLA has gained tremendous interests and attention due to the ever-increasing alarming issues such as resource sustainability and environment pollution caused by plastic wastes. Currently, crude oil is used both as the energy source for transportation and as the material source for chemical and plastic industries. However, the development of the petroleum industry has caused serious environmental issues, such as greenhouse gas emissions and global warming, which require immediate and drastic solutions. To resolve these issues, bio-degradable and bio-based plastics have been developed as a promising alternative to conventional plastics. In comparison to most other bio-based and bio-degradable plastics, PLA is by far the most important and promising one for rigid applications.

Its upper price as related with conventional petrochemical polymers was one of the biggest drawbacks that confined its usage in the previous years. Today, new synthesizing methods such as ring opening polymerization (ROP), with different catalysts such as Sn and Zn, have led to a significant decrease in its price than in former years. Supplementary academic and industrial attention should be paid to the development of new PLA preparing methods, which would be less costly and more complex than those of the other polymerization routes.

According to the studies reported in this review paper, PLA would be one of the most promising candidates for numerous industrial purposes. Its multiple desirable properties such as low cost, renewability, excellent biocompatibility, great materials assets, transparency and thermoplasticity open up many fields of application. Furthermore, since its discovery in 1932 by Carothers at DuPont, the alarming environmental issues mentioned above force us toward more extensive use of PLA. Nevertheless, PLA exhibits numerous limitations such as low heat stability, water barrier properties, oxygen permeability and mechanical properties. For these reasons, remarkable efforts have been made to enhance the performance of PLA by increasing its molecular weight via solid state and chain extending techniques. The type of used chain extender and pre-crystallization, catalyst, temperature and time in SSP are the most critical factors to produce high molecular weight PLA.

In the biomedical fields, PLA composites provide great solutions such as bone tissue engineering, the treatment of blood vessels, and organs and skin regeneration. PLA in the form of micro- and nano-particles loaded with different drugs can be used for tumor therapy and drug delivery applications. In terms of materials, the studies reviewed in this paper constitute PLA as a very reliable substance for constructions, automotive industry, packaging, agriculture, textiles, electronics along with single use items.

The current scope of the PLA industry is a great indication of the dominant position of PLA among other bioplastics. In addition, the ever-growing number of pilot projects announced or under construction, the numerous products and applications of PLA in bio-degradable and/or bio-based polymer markets, and the number of polymer companies and converters involved in PLA proves its significance.

To conclude, with all things considered, the protection of the environment is more critical nowadays than ever. In an attempt to totally replace petroleum-based plastics with biobased polymers, PLA seems as one of the most dominant succorers for this achievement. However, researchers and companies are forced to overcome the numerous challenges and limitations that are presented in its monomer and polymer synthesis as well as in its applications.

## Figures and Tables

**Table 1:** Typical results of the catalyst screening test for the melt polycondensation of OLLA Reprinted with permission from ref. [40]. Copyright 2000 John Wiley & Sons, Inc.

Run	Catalyst	Catalyst/OLLA (wt%)	Temperature (°C)	Time (h)	Pressure (torr)	MW (g/mol)	Yield (%)
1	GeO_2_	0.8	180	20	10	28,000	73
2	Sb_2_O_3_	0.1	200	30	20	20,000	25
3	ZnO	0.1	200	30	20	36,000	35
4	Fe_2_O_3_	0.1	200	8	1	20,000	23
5	Al_2_O_3_	8.5	200	30	20	27,000	42
6	SiO_2_	0.8	180	20	10	11,000	58
7	TiO_2_	0.8	180	20	10	11,000	64
8	SnO	0.2	180	20	10	50,000	36
9	SnCl_2_·2H_2_O	0.4	180	20	10	41,000	43
10	TSA	0.34	180	10	10	17,000	70

**Table 2:** Improvement in properties achieved and applications of PLA composites.

PLA/Composite	Improvement in Properties Achieved	Application	Refs.
PLA/ZnO membranes	Mechanical properties, UV, visiblelight barrier performances.	Food packaging	[125]
PLA/Chitosan/Basil olive oil films	Tensile, barrier, and antioxidant properties.	Active food packaging	[126]
PLA/poly(butylene succinate)/cellulose fiber composite	Viscosity, Thermal properties, crystallinity, flexural modulus.	Hot cups or lids application	[127]
PLA/lignin films	Toughness, antioxidant performance, water vapor barrier properties, antioxidant, UV resistance behavior	Active food packaging	[128]
PLA/poly(vinyl alcohol)/poly(ethylene glycol) blends/thyme essential oil	Hydrophilicity, antibacterial properties	Antimicrobial packaging	[129]
PLA/chitin/cellulose nanofiber	Mechanical, thermal, and wettability properties	Green packaging	[130]
PLA/lycopene nanocomposite film	Mechanical properties, oxidative and color stability of margarine	Margarine packaging	[131]
PLA/selenium microparticles films	Water resistance, ultraviolet resistance, antibacterial and oxidation resistance	Food packaging	[132]
PLA/silica aerogel composite	Crystallinity, stretchability, elongation at break, high transparency	Food packaging	[133]
PLA/halloysite nanotubes bionanocomposite	Thermal properties, mechanical properties (tensile strength, yield strength).	Food packaging	[122]
PLA/poly(butylene adipate-co-terephthalate) (PBAT) blends with incorporated trans-cinnamaldehyde	Antimicrobial properties, antifungal properties.	Bread packaging	[134]
PLA/cinnamaldehyde inclusions films	Antibacterial properties, tensile strength, water and oxygen resistance, life expectancy.	Fruit packaging	[123]
PLA/grape syrup films	UV absorption and light stability	Food packaging	[135]

**Table 3:** PLA composites and the improvements in properties achieved in textiles.

PLA/Composite	Improvement in Properties Achieved	Refs.
PLA/Ramie	PLA brittleness reduced Acceleration of PLA’s biodegradation rate Reduction of carbon footprint	[143]
PLA/CCF (continuous carbon fiber)	High tensile strength	[144]
PLA/Regenerated cellulose	Improved rheological characteristics	[145]
PLA/cotton fabric	Improved flexural properties	[146]

**Table 4:** Improvements of properties achieved in PLA reinforced with fibers.

PLA Fiber Reinforcement	Improvements in the Properties Achieved	Application	References
Alfa fibers	Tensile strength	Ecological and economical components	[149]
Basalt fibers	Mechanical properties	Excellent strength and fracture toughness components	[150]
Glass fibers	Mechanical properties, Thermal behavior, Foaming ability	Light weight structural components	[151]

**Table 5:** Key PLA Projects with estimated capacities in 2019–2020 [9].

Key PLA Projects in 2018–2020	Location of Operation	KT
NatureWorks	USA	150
Total Corbion PLA JV	Thailand	75
Hisun	China	45
BBCA & Galactic	China	40
COFCO	China	10
Hengtian	China	10
SuPLA	China	10
TongJieLiang	China	10
Synbra	Netherlands	5
TianRen	China	3
Futerro (under Galactic)	Belgium	1
Jiangxi KeYuan	China	1
Sulzer	Switzerland	<1
Pyramid (under Udhe)	Germany	<1
Other announced projects (e.g., XinNing, HongDa, TongBang, YouCheng)	China	N/A

## Data Availability

Data is contained within the article.
